# A Convenient Synthesis of (16*S*,20*S*)-3β-Hydroxy-5α-pregnane-20,16-carbolactam and Its *N*-alkyl Derivatives

**DOI:** 10.3390/molecules25102377

**Published:** 2020-05-20

**Authors:** Agnieszka Wojtkielewicz, Damian Pawelski, Przemysław Bazydło, Aneta Baj, Stanisław Witkowski, Jacek W. Morzycki

**Affiliations:** Faculty of Chemistry, University of Białystok, K. Ciołkowskiego 1K, 15-245 Białystok, Poland; d.pawelski@uwb.edu.pl (D.P.); bazydlo_przemyslaw@wp.pl (P.B.); aneta.baj@uwb.edu.pl (A.B.); wit@uwb.edu.pl (S.W.); morzycki@uwb.edu.pl (J.W.M.)

**Keywords:** lactams, reductive amination, spirostane degradation, steroids

## Abstract

A concise synthesis of (16*S*,20*S*)-3β-hydroxy-5α-pregnane-20,16-carbolactam from tigogenin via the corresponding lactone is described. The most efficient synthetic route consisted of the lactone ring-opening with aminoalane reagent followed by PDC or Dess-Martin oxidation. The oxo-amide obtained was subjected to cyclization with Et_3_SiH/TFA or Et_3_SiH/Bi(TfO)_3_. Alternately, the lactone was converted first to the oxo-acid, which was then subjected to the microwave-assisted reductive amination. *N*-Alkyl derivatives were also obtained in a similar way.

## 1. Introduction

**Figure 1:**
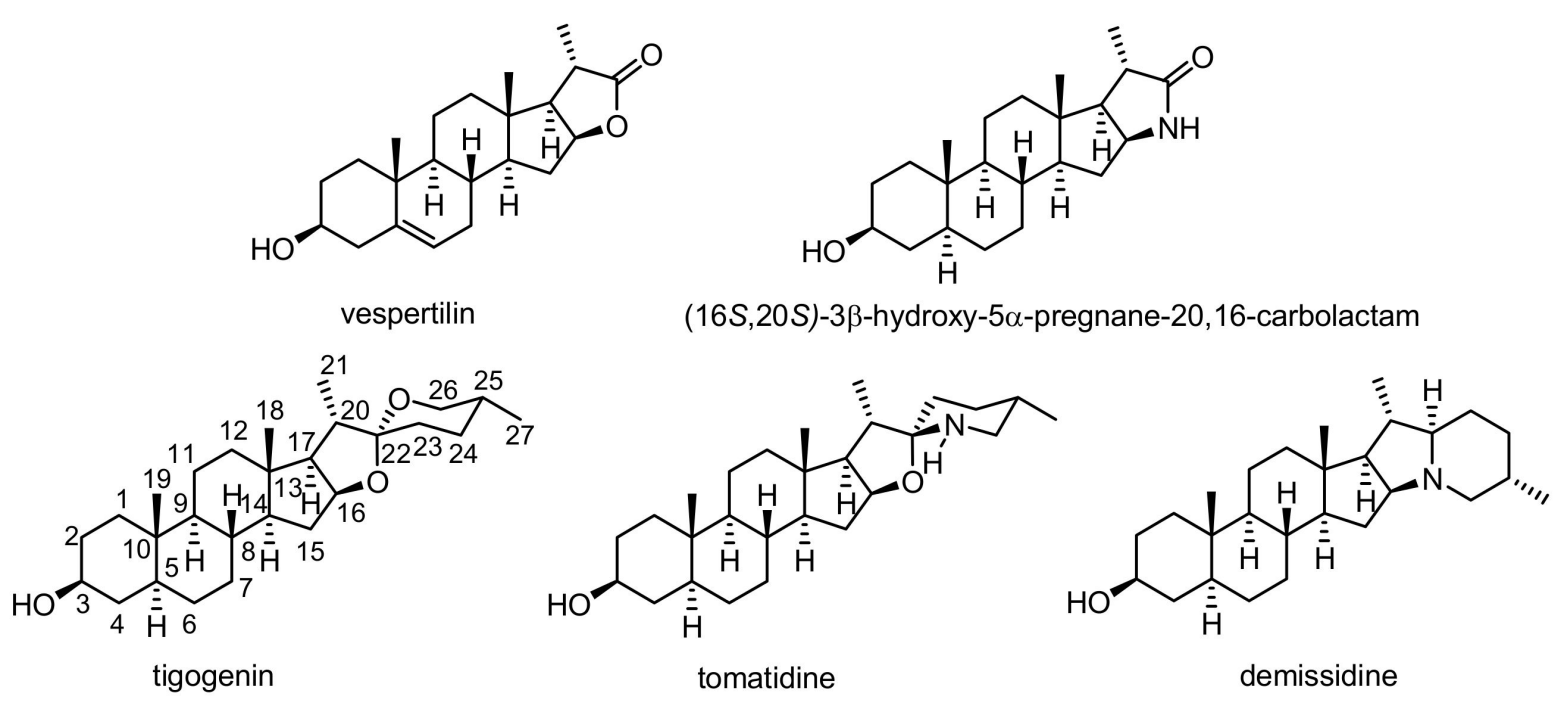
Vespertilin, the target lactam, tigogenin, and steroidal alkaloids.

In recent years, much attention has been paid to the preparation and biological activity evaluation of 23,24-bisnorcholano-22,16-lactones (bisnorcholanic lactones) [1]. One of them, (16*S*,20*S*)-3β-hydroxy-pregn-5-ene-20,16-carbolactone (vespertilin; Figure 1), is a natural product isolated from *Solanum vespertilio* [2]. Furthermore, other bisnorcholanic lactones have been isolated from different plants [3,4,5]. The lactones usually occur in plants as glycosides with sugar linked to the oxygen atom of aglycone at C3. Vespertilin shows a variety of biological activities including anticancer, antifungal, and bactericidal [6].

The availability of vespertilin and other bisnorcholanic lactones from natural sources is rather limited. However, they are readily available by degradation of steroidal sapogenins and alkaloids (spirosolanes, Figure 1). Alternately, bisnorcholanic lactones can be obtained from steroidal 17-ketones but the synthesis is long and expensive [7]. More conveniently, the lactones can be prepared by oxidation of steroidal spirostanes or spirosolanes either directly or via intermediate 23-oxo derivatives. The latter are readily available from sapogenins using the Barton’s procedure (NaNO_2_ in the presence of BF_3_·Et_2_O in glacial acetic acid) [8,9]. Further oxidation of 23-oxo-sapogenins to bisnorcholanic lactones can be easily carried out using different oxidizing agents, e.g., MCPBA/BF_3_·Et_2_O [10,11,12], H_2_O_2_/H^+^ [6], (PhSe)_2_/PhIO_2_ [13], or even may proceed with TMSOTf in the absence of an oxidant [14]. However, direct transformation of sapogenins or spirosolanes to bisnorcholanic lactones is advantageous over two-step procedures. Though spirosolanes (e.g., solasodine, tomatidine) have been recently reported [15,16] to directly yield lactones when treated with different oxidizing agents, they are more expensive than sapogenins. A practical and direct degradation method of tigogenin to the corresponding bisnorcholanic lactone has been patented by Chinese chemists [17,18]. The method consists of peroxyacid/I_2_ oxidation in acid medium. We have slightly improved the method by using more concentrated hydrogen peroxide/trifluoroacetic acid, and the new protocol is described in Supplementary Materials.

In contrast to the well-known bisnorcholanic lactones, the corresponding lactams, to the best of our knowledge, have not been reported in the literature yet. Many biologically active lactams have been isolated from natural sources and some of them are used, along with different synthetic lactam compounds, in contemporary medicine [19,20,21,22,23,24,25,26]. Herein, we report the results of our study on the first synthesis of (16*S*,20*S*)-3β-hydroxy-5α-pregnane-20,16-carbolactam and its *N*-alkyl derivatives (bisnorcholanic lactams).

## 2. Results and Discussion

**Scheme 1:**
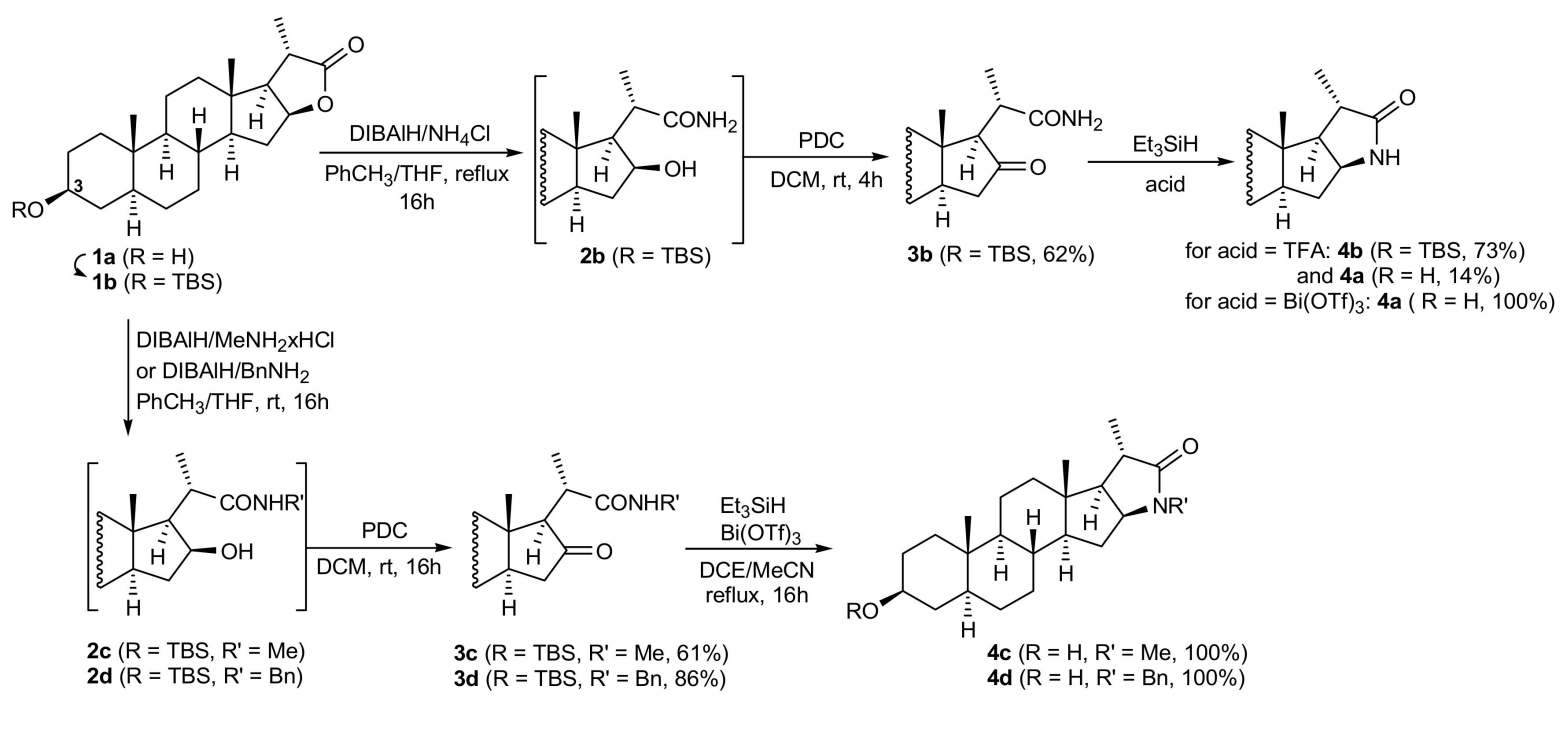
Synthesis of bisnorcholanic lactam derivatives via oxo-amide intermediates.

The first three-step strategy for the synthesis of the target lactam from bisnorcholanic lactone involves lactone ammonolysis to corresponding hydroxy-amide, its oxidation to oxo-derivative, followed by reductive cyclization (Scheme 1).

Our study began from an improvement of Chinese method of the lactone synthesis from tigogenin [17,18]. The use of a 60% solution of hydrogen peroxide and a catalytic amount of trifluoroacetic acid increased the yield to 91% on the multigram scale (see Supplementary Materials). With a sufficient amount of protected lactone **1b** in hand, we tried the ammonolysis reaction (Scheme 1). The classical lactone ammonolysis with gaseous NH_3_ or ammonia aqueous solution in MeOH/THF [27], as well as a microwave assisted reaction [28], failed in the case of the bisnorcholanic lactone **1b**. Therefore, we employed aminoalane reagent prepared in situ from DIBAlH and ammonium chloride. In our previous studies, we discovered that it is a very efficient reagent for amide and nitrile synthesis from acids and their derivatives [29]. The reaction of lactone **1b** with 20 equivalents of aluminum amide in THF at reflux provided the desired hydroxy-amide **2b** in high yield (Scheme 1). The product appeared to be unstable, when left neat or in solution in a freezer for 24 h, it underwent lactonization spontaneously. For that reason, crude **2b**, without purification, was subjected to oxidation. The oxidation of hydroxy-amide **2b** with various oxidizing reagents (Dess-Martin periodinane, PCC/AcONa, PDC, Swern reagent) was studied. The best yield (62% after two steps) of the desired oxo-derivative **3b** was obtained with PDC in dichloromethane (Scheme 1). The key step of the lactam synthesis was the cyclization of the obtained oxo-amide **3b** to the desired γ-lactam. Few methods of amide/lactam preparation by condensation of ketone and amide have been reported in the literature. Most of them are based on a two-step protocol comprising of inter- or intramolecular reactions of ketone and amide under acidic conditions, followed by enamide hydrogenation in the presence of rhodium or ruthenium catalysts [30,31,32,33]. Among the reported procedures, there is also a reductive cyclization method leading directly to lactam. Frequently triethylsilane in presence of acid was used for the transformation of oxo-amide into lactam [34,35]. Another convenient reagent for this transformation is sodium triacetoxyborohydride in acetic acid [36]. After reviewing the literature, we decided to subject the oxo-amide **3b** to simultaneous cyclization and ionic hydrogenation with EtSiH and TFA. The reaction of compound **3b** with 20 equivalents of EtSiH and 10 equivalents of TFA in refluxing DCE resulted in the desired lactam in high yield. However, the partial deprotection of TBS ether was observed under the employed conditions. Therefore, the 3-TBS protected lactam **4b** was isolated in 73% yield in addition to the corresponding desilylated lactam **4a** (14%). Decreasing either the amount of acid used or the reaction temperature did not prevent the partial 3-TBS hydrolysis. However, when bismuth triflate or TBS triflate were employed instead of TFA for the reductive cyclization, only the deprotected product **4a** was isolated quantitatively.

**Figure 2:**
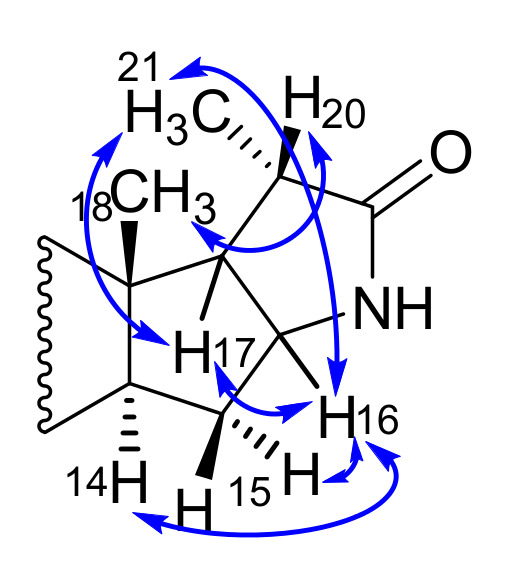
NOE and ROESY correlations diagram for compounds **4a.**

The configurations at C16, C17, and C20 stereogenic centers remained intact during the lactam synthesis as proved by NOE and ROESY correlation experiments (Figure 2). The differential NOE as well as ROESY spectra obtained for compound **4a** showed a strong correlation between 17-H at 1.82 ppm (dd) with 16-H at 4.02 ppm (m) and 21-CH_3_ protons at 1.21 ppm (d), as well as a correlation between 18-methyl protons at 0.75 ppm (s) and 20-H at 2.34 (d), and no correlation between 18-CH_3_ and 17-H at 1.82 ppm (dd). This implied 16α, 17α, and 20β proton configurations. These configuration assignments were confirmed by correlations of 16-H at 4.02 ppm (m) with 21-methyl protons at 1.21 ppm (d) and 14α-H at 1.05 ppm (m) (Figure 2) observed in ROESY spectrum.

The described methodology for bisnorcholanic lactam synthesis was successfully applied to preparation of its *N*-alkyl derivatives (Scheme 1, lower row). In the case of *N*-substituted lactams, the first step included lactone **1b** aminolysis with aminoalane reagent prepared from DIBAlH and an appropriate amine or its hydrochloride affording secondary hydroxy-amides. The reactions of *N*-alkylaminoalanes with lactone **1b** proceeded smoothly at room temperature. Employing the reported procedure, *N-*methyl and *N-*benzyl derivatives **2c** and **2d** were prepared. The further syntheses of *N*-alkyl-lactams from the secondary amides **2c** and **2d** were carried out analogously to their *N*-unsubstituted congener. Although the secondary amides (**2c**, **2d**) appeared to be less prone to lactonization than the analogous primary hydroxy-amide **2b**, they underwent spontaneous intramolecular alcoholysis when allowed to stand for a longer period of time. Therefore, they were oxidized without purification with PDC to provide the desired oxo-derivatives **3c** and **3d** in high yields. The reductive cyclization with EtSiH/Bi(OTf)_3_ produced target *N*-alkylated lactams **4c** and **4d** quantitatively (61% and 86% total yields, respectively, for the three-step synthesis from lactone).

**Scheme 2:**
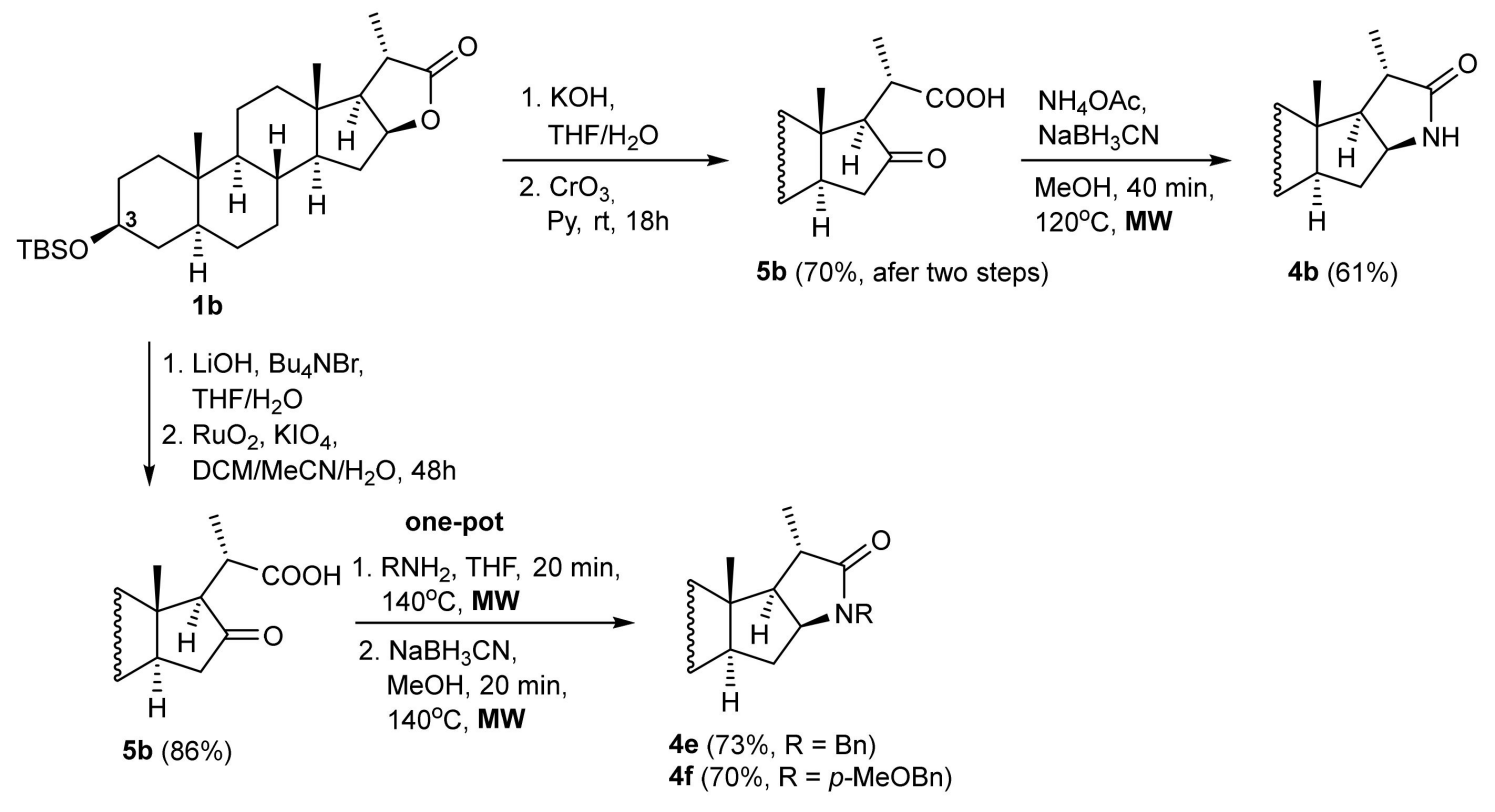
Synthesis of bisnorcholanic lactam derivatives via an oxo-acid intermediate.

Taking into account the economic aspect of bisnorcholanic lactam synthesis, we also tested other synthetic strategies. The second approach involved the saponification of the tigogenin derived lactone, followed by oxidation of the obtained hydroxy-acid to oxo-acid and reductive amination. It was expected that the γ-amino acid should spontaneously cyclize to the corresponding γ-lactam under neutral conditions. A base induced ring-opening of lactone **1b** resulted in the formation of the corresponding hydroxy-acid, which rapidly cyclized in solution at room temperature back to the lactone. To avoid lactonization, the crude hydroxy-acid was immediately oxidized to oxo-acid **5b** (Scheme 2). From several tested oxidizing agents (PDC/DCM, Dess Martin, CrO_3_) the best result was obtained using CrO_3_ in pyridine (70% after two steps). Although the literature describes a cyclic form of oxo-acid (16α-hydroxy-5α-pregnane-20,16-carbolactone) [37], we cannot confirm the reported equilibrium of oxo-acid **5b** with its cyclic form. In addition to the expected product **5b**, lactone **1b** was recovered in small amounts (15–20%). In order to avoid partial lactonization, basic conditions were employed for oxidation of the hydroxy-carboxylate initially formed during hydrolysis. In the first attempt, a one-pot protocol was employed for hydrolysis and oxidation. The obtained hydrolysis product, γ-hydroxy-carboxylate, was treated in situ with an aqueous solution of sodium hypochlorite (NaOCl). After a two-day reaction at room temperature, only a minor conversion of γ-hydroxy-carboxylate to the oxo derivative was observed. After changing of the oxidizing agent to RuO_2_/KIO_4_ in water-MeCN-DCM biphasic system, [38] the desired oxo-acid **5b** was obtained in 85% yield. This intermediate was transformed directly into lactam by reductive amination followed by spontaneous cyclization. A large number of reducing hydride reagents have been studied for direct reductive amination, including NaBH_3_CN [39], NaBH(OAc)_3_ [40], pyridine–BH_3_ [41], and NaBH_4_/titanium(IV) isopropoxide [42]. In our study, the classical Leuckart reaction [NH_4_OCHO] as well as the reductive amination with ammonium acetate [NH_4_OAc (10–15 equiv.), NaBH_3_CN (1–5 equiv.) in MeOH or MeOH/THF] [43] proved unsuccessful even at elevated temperature. The addition of acid resulted in a complex mixture of products with lactone **1b** in the largest amount [44]. Finally, the application of microwaves (MW) [45] for reductive amination/cyclization generated the expected lactam **4b** in 61% yield from oxo-acid **5b** (52% total yield from lactone **1b**). The use of a decreased amount of NaBH_3_CN (1.2–3 equiv.) prevented the formation of lactone **1b** as a by-product. The elaborated alternative method, like the previously described one, proved also be a convenient way for preparation *N*-alkylated derivatives of lactam **4b**. We examined the reductive amination of oxo-acid **5b** under MW irradiation in the presence of amines such as BnNH_2_ and *p*MeOBnNH_2._ In this case, the best results were obtained in a one-pot two-step synthesis. The reducing agent was added to the reaction mixture when the imine formation was completed. An increase of NaBH_3_CN excess from 1 to 3 equiv. provided higher product yield. The microwave assisted reductive amination/cyclization generated the *N*-substituted lactams from oxo-acid **5b** in a short reaction time (30–40 min at 140 °C) and satisfactory yields (70–73%). Target lactams **4e** and **4f** were obtained from lactone **1b** in 63% and 60% total yield, respectively.

## 3. Materials and Methods

### 3.1. General

NMR spectra were recorded with Bruker Avance II 400 spectrometer operating at 400 MHz, using CDCl_3_ solutions with TMS as the internal standard (only selected signals in the ^1^H NMR spectra are reported). Coupling constants (*J*) are given in Hz. The FTIR spectra were obtained using Nicolet™ 6700 spectrometer (Thermo Scientific, Waltham, MA, USA). The spectra were recorded in the range between 4000 and 500 cm^−1^ with a resolution of 4 cm^−1^ and 32 scans using Attenuated Total Reflectance (ATR) techniques. ESI and ESI-HRMS spectra were obtained on the Agilent 6530 Accurate-Mass Q-TOF ESI and LC/MS system. Melting points were determined using MP70 Melting Point System (Mettler Toledo, Greifensee, Switzerland). Microwave reactions were performed in a Discover SP microwave synthesizer (CEM Corp., Matthews, NC, USA) in a closed vessel with maximum power input of 300 W. Thin-layer chromatography (TLC) was performed on aluminum plates coated with silica gel 60 F254 (Merck, Darmstadt, Germany), by spraying with ceric ammonium molybdate (CAM) solution, followed by heating. The reaction products were isolated by column chromatography, performed using 70–230 mesh silica gel (J.T. Baker, Center Valley, PA, USA). Tigogenin was obtained by hydrogenation of commercial diosgenin (Sigma-Aldrich) in presence of Pd/C catalyst [46].

### 3.2. Chemical Synthesis

#### 3.2.1. Synthesis of Bisnorcholanic Lactam Derivatives via Oxo-Amide Intermediates

##### Procedure for Lactam **4a** and **4b** Synthesis

Preparation of the Aminoalane Reagent from DIBALH and NH_4_Cl.

A solution of DIBAlH in toluene (1 M, 2.17 mL, 2.17 mmol, 20 equiv. relative to lactone **1b**) was added to a cooled (0–5 °C) suspension of NH_4_Cl (0.122 g, 2.28 mmol, 21 equiv.) in anhydrous THF (10 mL) under argon. The reaction was stirred for 15 min in an ice bath and then 1.5 h at room temperature. After this time, the obtained reagent solution was used directly for amide synthesis.

##### Synthesis of Oxo-Amide **3b**

The solution of aminoalane reagent (prepared from 20 equiv. of DIBAlH) was added dropwise to a solution of lactone **1b** (0.05 g, 0.1087 mmol, 1 equiv.) in anhydrous THF (ca. 6 mL) at room temperature. Stirring was continued for 16 h at reflux. After this time, the reaction mixture was cooled, quenched with aqueous solution of KHSO_4_ and the product was extracted with ether. The extract was washed with water, dried over anhydrous sodium sulfate, and the solvent was evaporated. The crude product **2b** (^1^H NMR (CDCl_3_, 400 MHz) δ 5.73 (bs, 1H), 5.31 (bs, 1H), 4.29 (m, 1H), 3.55 (m, 1H), 2.96 (m, 1H), 2.83 (m, 1H), 2.22 (m, 1H), 1.90 (m, 1H), 1.26 (d, *J* = 6.8, 3H), 0.91 (s, 3H), 0.89 (s, 9H), 0.82 (s, 3H), 0.06 (s, 6H)) was immediately oxidized. PDC (3 equiv., 0.336 mmol, 0.123 g) was added to the solution of crude hydroxyamide **2b** in dry DCM. The reaction was stirred for 3 h at room temperature. The solvent was evaporated and the crude product was purified by silica gel column chromatography with MeOH/DCM (1:99) elution. Product **3b** was obtained in 62% yield (after two steps).

(20*S*)-3β-*t*-butyldimetylsilyloxy-16-oxo-5α-pregnane-20-carboxyamide (**3b**): white solid m.p. 210–212 °C (DCM/MeOH). ^1^H NMR (CDCl_3_, 400 MHz) δ 5.81 (bs, 1H), 5.50 (bs, 1H), 3.56 (m, 1H), 2.48 (d, *J* = 9.9, 1H), 2.34 (m, 1H), 2.22 (m, 1H), 2.01 (m, 1H), 1.22 (d, *J* = 7.0, 3H), 0.88 (s, 9H), 0.81 (s, 3H), 0.76 (s, 3H), 0.04 (s, 6H). ^13^C NMR (CDCl_3_, 100 MHz) δ, 218.4 (C), 179.0 (C), 72.0 (CH), 65.3 (CH), 54.1 (CH), 50.8 (CH), 44.9 (CH), 42.4 (C), 38.84 (CH), 38.82 (CH_2_), 38.5 (CH_2_), 37.7 (CH_2_), 36.8 (CH_2_), 35.6 (C), 34.4 (CH), 32.1 (CH_2_), 31.8 (CH_2_), 28.5 (CH_2_), 25.9 (3CH_3_), 20.7 (CH_2_), 18.3 (C), 17.1 (CH_3_), 13.1 (CH_3_), 12.3 (CH_3_), −4.6 (2CH_3_). IR (ATR): ν_max_ (cm^−1^): 3400, 3194, 1733, 1700, 1635, 1459, 1250, 1097, 1085. ESI-MS 498 [M + Na]^+^, 974 [2M + Na]^+^. HRMS calcd. for C_28_H_49_NO_3_SiNa 498.3374 (M + Na)^+^, found 498.3364.

##### Synthesis of Lactam **4b** with EtSiH/TFA

TFA (0.048 mL, 0.63 mmol, 10 equiv.) and Et_3_SiH (0.2 mL, 1.26 mmol, 20 equiv.) were added to the solution of oxo-amide **3b** (30 mg, 0.063 mmol) in dry DCE (4 mL). The reaction mixture was stirred for 16 h at reflux. Then it was poured into water and product was extracted by DCM. The extract was dried over anhydrous sodium sulfate, and the solvent was evaporated. Silica gel column chromatography afforded two products: 3-TBS lactam **4b** (73%) eluted with 0.7% MeOH/DCM and 3-hydroxy-lactam **4a** (14%) eluted with 3% MeOH/DCM.

(16*S*,20*S*)-3β-*t*-butyldimetylsilyloxy-5α-pregnane-20,16-carbolactam (**4b**): white crystals, m.p. 275–277 °C (DCM/MeOH). ^1^H NMR (CDCl_3_, 400 MHz) δ 5.72 (bs, 1H), 4.05 (m, 1H), 3.55 (m, 1H), 2.37 (m, 1H), 2.09 (m, 1H), 1.24 (d, *J* = 7.4, 3H), 0.89 (s, 9H), 0.81 (s, 3H), 0.78 (s, 3H), 0.05 (s, 6H). ^13^C NMR (CDCl_3_, 100 MHz) δ, 181.7 (C), 72.0 (CH), 58.9 (CH), 55.4 (CH), 55.3 (CH), 54.6 (CH), 45.0 (CH), 42.0 (C), 38.8 (CH_2_), 38.6 (CH_2_), 37.2 (CH_2_), 36.7 (CH), 35.6 (C), 35.0 (CH), 33.9 (CH_2_), 32.3 (CH_2_), 31.9 (CH_2_), 28.6 (CH_2_), 25.9 (3CH_3_), 20.6 (CH_2_), 18.7 (C), 18.2 (CH_3_), 14.3 (CH_3_), 12.4 (CH_3_), -4.6 (2CH_3_). IR (ATR): ν_max_ (cm^−1^): 3173, 3082, 1701, 1655, 1454, 1249, 1102. ESI−MS 460 [M + H]^+^, 941 [2M + Na]^+^. HRMS calcd. for C_28_H_50_NO_2_Si 460.3605 (M + H)^+^, found 460.3598.

##### Synthesis of Lactam **4a** with EtSiH/Bi(OTf)_3_

Bi(OTf)_3_ (14 mg, 0.5 equiv.) and Et_3_SiH (0.012 mL, 2 equiv.) were added to the solution of oxo-amide **3b** (20 mg, 0.04 mmol) in dry DCE (2 mL)/MeCN (2 mL). The reaction mixture was stirred for 16 h at reflux. After cooling the reaction mixture was poured into water and product was extracted with chloroform. The extract was dried over anhydrous sodium sulfate, and the solvent was evaporated. Silica gel column chromatography yield quantitatively product **4a** eluted with 3% MeOH/DCM.

(16*S*,20*S*)-3β-hydroxy-5α-pregnane-20,16-carbolactam (**4a**): white crystals, m.p. 210–212 °C (DCM/MeOH). ^1^H NMR (CDCl_3_, 400 MHz) δ 6.53 (bs, 1H), 4.02 (m, 1H), 3.56 (m, 1H), 2.34 (m, 1H), 2.23 (bs, 1H), 2.06 (m, 1H), 1.21 (d, *J* = 7.5, 3H), 0.80 (s, 3H), 0.75 (s, 3H). ^13^C NMR (CDCl_3_, 100 MHz) δ, 182.0 (C), 71.0 (CH), 58.7 (CH), 55.6 (CH), 55.1 (CH), 54.4 (CH), 44.7 (CH), 41.9 (C), 38.7 (CH_2_), 38.0 (CH_2_), 36.9 (CH_2_), 36.8 (CH), 35.5 (C), 34.9 (CH), 33.7 (CH_2_), 32.1 (CH_2_), 31.93 (CH_2_), 28.5 (CH_2_), 20.6 (CH_2_), 18.6 (CH_3_), 14.2 (CH_3_), 12.3 (CH_3_). IR (ATR): ν_max_ (cm^−1^): 3267, 1685, 1450, 1044. ESI−MS 346 [M + H]^+^, 713 [2M + Na]^+^. HRMS calcd. for C_22_H_36_NO_2_ 346.2741 (M + H)^+^, found 346.2742.

##### Procedure for N-Alkyl-Lactam **4c** and **4d** Synthesis

Preparation of aminoalane reagent from DIBALH and BnNH_2_ or MeNH_2_xHCl.

The desired aminoalane reagents were prepared from solution of DIBALH in toluene (1 M, 10 equiv. with reference to lactone **1b**, 2.17 mmol, 2.17 mL) and MeNH_2_xHCl (10.3 equiv. 2.2 mmol, 0.15 g) or BnNH_2_ (10.3 equiv. 2.2 mmol, 0.24 mL), analogously to aminoalane preparation from DIBALH and NH_4_Cl.

##### Synthesis of Hydroxy-Amides **2c** and **2d**

The solution of aminoalane reagent (prepared from 10 equiv. of DIBAlH) was added dropwise to a solution of lactone **1b** (0.1 g, 0.217 mmol, 1 equiv.) in anhydrous THF (ca 4 mL) at room temperature. Stirring was continued for 16 h at rt. After this time, the reaction mixture was cooled, quenched with aqueous solution of KHSO_4_ and the product was extracted with ether. The extract was washed with water, dried over anhydrous sodium sulfate, and the solvent was evaporated. The crude product, without purification, was used in the next step.

(20*S*)-*N*-methyl-3β-*t*-butyldimetylsilyloxy-16β-hydroxy-5α-pregnane-20-carboxyamide (**2c**): white solid, ^1^H NMR (CDCl_3_, 400 MHz) δ 5.72 (d, *J* = 4.5, 1H), 1H), 4.21 (m, 1H), 3.55 (m, 1H), 3.42 (bs, 1H), 2.83 (d, *J* = 4.8, 3H), 2.19 (m, 1H), 1.89 (m, 1H), 1.23 (d, *J* = 7.0, 3H), 0.90 (s, 3H), 0.89 (s, 9H), 0.81 (s, 3H), 0.05 (s, 6H). ^13^C NMR (CDCl_3_, 100 MHz) δ, 178.5 (C), 72.5 (CH), 72.1 (CH), 59.7 (CH), 54.4 (CH), 54.2 (CH), 45.0 (CH), 42.7 (C), 40.2 (CH_2_), 38.8 (CH), 38.6 (CH_2_), 37.1 (CH_2_), 35.8 (CH_2_), 35.5 (C), 35.1 (CH), 32.0 (CH_2_), 31.9 (CH_2_), 28.6 (CH_2_), 26.4 (CH_3_), 25.9 (3CH_3_), 20.9 (CH_2_), 18.3 (C), 16.6 (CH_3_), 13.2 (CH_3_), 12.4 (CH_3_), −4.6 (2CH_3_). ESI−MS 492 [M + H]^+^. HRMS calcd. for C_29_H_54_NO_3_Si 492.3873 (M + H)^+^, found 492.3888.

(20*S*)-*N*-benzyl-3β-*t*-butyldimetylsilyloxy-16β-hydroxy-5α-pregnane-20-carboxyamide (**2d**): white solid, ^1^H NMR (CDCl_3_, 400 MHz) δ 7.32 (m, 3H), 7.28 (m, 2H), 6.09 (t, *J* = 5.7, 1H), 4.45 (d, *J* = 5.7, 2H), 4.22 (m, 1H), 3.55 (m, 1H), 2.83 (m, 1H), 2.19 (m, 1H), 1.89 (m, 1H), 1.25 (d, *J* = 7.1, 3H), 0.89 (s, 12H), 0.81 (s, 3H), 0.06 (s, 6H). ^13^C NMR (CDCl_3_, 100 MHz) δ, 177.6 (C), 138.4 (C), 128.7 (2CH), 127.7 (2CH), 127.5 (CH), 72.4 (CH), 72.1 (CH), 59.4 (CH), 54.5 (CH), 54.1 (CH), 44.9 (CH), 43.5 (CH_2_), 42.7 (C), 40.1 (CH_2_), 38.9 (CH), 38.6 (CH_2_), 37.1 (CH_2_), 36.0 (CH_2_), 35.5 (C), 35.1 (CH), 32.0 (CH_2_), 31.9 (CH_2_), 28.6 (CH_2_), 25.9 (3CH_3_), 20.9 (CH_2_), 18.2 (C), 16.7 (CH_3_), 13.2 (CH_3_), 12.3 (CH_3_), −4.6 (2CH_3_). ESI−MS 568 [M + H]^+^, 1157 [2M + Na]^+^. HRMS calcd. for C_35_H_58_NO_3_Si 568.4186 (M + H)^+^, found 568.4197.

##### Synthesis of Oxo-Amides **3c** and **3d**

PDC (4 equiv.) was added to the solution of crude hydroxy-amide **2c** or **2d** in dry DCM. The reaction was stirred for 16 h at room temperature. The solvent was evaporated and the crude product was purified by silica gel column chromatography. Product **3c** was obtained in 61% yield (after two steps) with hexane/AcOEt (3:7) elution. Product **3d** was obtained in 86% yield (after two steps) with hexane/AcOEt (75:25) elution.

(20*S*)-*N*-methyl-3β-*t*-butyldimetylsilyloxy-16-oxo-5α-pregnane-20-carboxyamide (**3c**): white crystals, m.p. 203–204 °C (hexane/EtOAc). ^1^H NMR (CDCl_3_, 400 MHz) δ 5.63 (bd, *J* = 4.8, 1H), 3.55 (m, 1H), 2.84 (d, *J* = 4.8, 3H), 2.51 (d, *J* = 9.8, 1H), 2.23 (m, 2H), 2.02 (dt, *J*_1_ = 2.8, *J*_2_ = 9.3, 1H), 1.21 (d, *J* = 7.0, 3H), 0.89 (s, 9H), 0.83 (s, 3H), 0.76 (s, 3H), 0.06 (s, 6H). ^13^C NMR (CDCl_3_, 100 MHz) δ, 218.5 (C), 177.2 (C), 72.0 (CH), 65.2 (CH), 54.0 (CH), 50.7 (CH), 44.8 (CH), 42.4 (C), 39.6 (CH), 38.8 (CH_2_), 38.5 (CH_2_), 37.8 (CH_2_), 36.8 (CH_2_), 35.6 (C), 34.4 (CH), 32.1 (CH_2_), 31.8 (CH_2_), 28.4 (CH_2_), 26.4 (CH_3_), 25.9 (3CH_3_), 20.7 (CH_2_), 18.3 (C), 17.1 (CH_3_), 13.1 (CH_3_), 12.3 (CH_3_), −4.6 (2CH_3_). IR (ATR): ν_max_ (cm^−1^): 3294, 1739, 1643, 1558, 1461, 1250, 1099. ESI−MS 490 [M + H]^+^. HRMS calcd. for C_29_H_52_NO_3_Si 490.3716 (M + H)^+^, found 490.3725.

(20*S*)-*N*-benzyl-3β-*t*-butyldimetylsilyloxy-16-oxo-5α-pregnane-20-carboxyamide (**3d**): white crystals, m.p. 199–200 °C (hexane/EtOAc). ^1^H NMR (CDCl_3_, 400 MHz) δ 7.36 (m, 4H), 7.28 (m, 1H), 5.87 (t, *J* = 5.5, 1H), 4.64 (dd, *J*_1_ = 5.5, *J*_2_ = 14.8, 1H), 4.44 (dd, *J*_1_ = 5.5, *J*_2_ = 14.8, 1H), 3.57 (m, 1H), 2.58 (d, *J* = 10.0, 1H), 2.25 (m, 2H), 2.03 (m, 1H), 1.24 (d, *J* = 7.0, 3H), 0.90 (s, 9H), 0.83 (s, 3H), 0.76 (s, 3H), 0.07 (s, 6H). ^13^C NMR (CDCl_3_, 100 MHz) δ, 218.3 (C), 176.3 (C), 138.6 (C), 128.6 (2CH), 127.8 (2CH), 127.3 (CH), 72.0 (CH), 65.2 (CH), 54.0 (CH), 54.5 (CH), 50.7 (CH), 44.9 (CH), 43.7 (CH_2_), 42.4 (C), 39.8 (CH), 38.9 (CH_2_), 38.5 (CH_2_), 37.7 (CH_2_), 36.8 (CH_2_), 35.6 (C), 34.4 (CH), 32.1 (CH_2_), 31.8 (CH_2_), 28.5 (CH_2_), 25.9 (3CH_3_), 20.7 (CH_2_), 18.2 (C), 17.2 (CH_3_), 13.1 (CH_3_), 12.3 (CH_3_), −4.6 (2CH_3_). IR (ATR): ν_max_ (cm^−1^): 3280, 1737, 1640, 1557, 1451, 1250, 1100. ESI−MS 566 [M + H]^+^, 1153 [2M + Na]^+^. HRMS calcd. for C_35_H_56_NO_3_Si 566.4029 (M + H)^+^, found 566.4046.

##### Procedure for Lactam **4c** and **4d** Synthesis

Bi(OTf)_3_ (0.5 equiv.) and Et_3_SiH (2 equiv.) were added to the solution of oxo-amide **3c** or **3d** (10 mg) in mixture of dry DCE (2 mL) and MeCN (2 mL). The reaction mixture was stirred for 16 h at reflux. Then the reaction mixture was poured into water and product was extracted by DCM. The extract was dried over anhydrous sodium sulfate, and the solvent was evaporated. The crude product was purified by silica gel column chromatography. Product **4c** was eluted with hexane/AcOEt (2:8). Product **4d** was eluted with hexane/AcOEt (1:1).

(16*S*,20*S*)-*N*-methyl-3β-hydroxy-5α-pregnane-20,16-carbolactam (**4c**): obtained in quantitative yield as an amorphous solid. ^1^H NMR (CDCl_3_, 400 MHz) δ 3.92 (m, 1H), 3.55 (m, 1H), 2.76 (s, 3H), 2.42 (q, *J* = 7.4, 1H), 2.01 (ddd, *J*_1_ = 5.9, *J*_2_ = 7.8, *J*_3_ = 12.0, 1H), 1.20 (d, *J* = 7.4, 3H), 0.82 (s, 3H), 0.68 (s, 3H). ^13^C NMR (CDCl_3_, 100 MHz) δ, 178.1 (C), 71.2 (CH), 61.9 (CH), 55.9 (CH), 54.7 (CH), 54.4 (CH), 44.8 (CH), 41.9 (C), 38.8 (CH_2_), 38.1 (CH_2_), 37.04 (CH), 36.98 (CH_2_), 35.6 (C), 35.0 (CH), 32.2 (CH_2_), 31.5 (CH_2_), 31.1 (CH_2_), 28.5 (CH_2_), 27.6 (CH_3_), 20.6 (CH_2_), 18.8 (CH_3_), 14.2 (CH_3_), 12.3 (CH_3_). IR (ATR): ν_max_ (cm^−1^): 3329, 1661, 1448, 1051. ESI−MS 360 [M + H]^+^, 741 [M + Na]^+^. HRMS calcd. for: C_23_H_38_NO_2_ 360.2903 (M + H)^+^, found 360.2906.

(16*S*,20*S*)-*N*-benzyl-3β-hydroxy-5α-pregnane-20,16-carbolactam (**4d**): obtained in quantitative yield as an amorphous solid. ^1^H NMR (CDCl_3_, 400 MHz) δ 7.32 (m, 3H), 7.22 (m, 2H), 5.02 (d, *J* = 14.5, 1H), 3.81 (d, *J* = 14.5, 1H), 3.75 (m, 1H), 3.59 (m, 1H), 2.50 (q, *J* = 7.4, 1H), 1.22 (d, *J* = 7.4, 3H), 0.81 (s, 3H), 0.72 (s, 3H). ^13^C NMR (CDCl_3_, 100 MHz) δ, 178.2 (C), 136.6 (C), 128.6 (2CH), 128.2 (2CH), 127.5 (CH), 71.2 (CH), 58.8 (CH), 55.6 (CH), 54.8 (CH), 54.4 (CH), 44.8 (CH), 44.3 (CH_2_), 42.0 (C), 38.8 (CH_2_), 38.1 (CH_2_), 37.1 (CH), 37.0 (CH_2_), 35.6 (C), 35.0 (CH), 32.2 (CH_2_), 31.5 (CH_2_), 31.1 (CH_2_), 28.5 (CH_2_), 20.6 (CH_2_), 19.0 (CH_3_), 14.3 (CH_3_), 12.3 (CH_3_). IR (ATR): ν_max_ (cm^−1^): 3359, 1659, 1441, 1046. ESI−MS 436 [M + H]^+^, 893 [2M + Na]^+^. HRMS calcd. C_29_H_42_NO_2_ 436.3216 (M + H)^+^, found 436.3224.

#### 3.2.2. Synthesis of Bisnorcholanic Lactam Derivatives via Oxo-Acid **5b**

##### Procedure for Oxo-Acid **5b** Synthesis

*Method I.* To a solution of lactone **1b** (1.0 g, 2.17 mmol, 1.0 equiv.), in THF (75 mL) a solution of KOH (0.608 g, 10.85 mmol, 5.0 equiv.) in water (30 mL) was added. The resulting suspension was stirred vigorously at 50 °C until a conversion of 100% was reached. The progress of the reaction was monitored by TLC (hexane/AcOEt, 7:3). After cooling to rt 1M hydrochloric acid was added until pH 6-7 was reached and the product was extracted with Et_2_O (3 × 50 mL). The combined organic layers were washed with brine, dried over anhydrous sodium sulfate, and concentrated under reduced pressure. Crude hydroxy-acid in dry pyridine was added to an ice-cold solution of CrO_3_ (0.428 g, 4.28 mmol, 2 equiv.) in pyridine (20 mL) and stirred at rt overnight. Then the mixture was decanted from the tarry residue, which was washed with three portions of diethyl ether. The combined organic solution was washed with 5% hydrochloric acid, aqueous 5% sodium hydrogen carbonate and brine. The obtained solution was dried over anhydrous sodium sulfate and the solvent was evaporated under reduced pressure. Purification by silica gel column chromatography gave lactone **1b** (20%) eluted with 70% hexane/AcOEt and oxo-acid **5b** (70%) eluted with 50% hexane/AcOEt.

*Method II.* To a solution of lactone **1b** (5.0 g, 0.011 mol, 1.0 equiv.), in THF/H_2_O (200 mL, 5/2) *n*-Bu_4_NBr (0.07 g, 2 mol%) and LiOH (1.3 g, 0.054 mol, 5.0 equiv.) were added. The resulting suspension was stirred overnight at room temperature. The progress of the reaction was monitored by TLC. After the hydrolysis was completed the solvent was evaporated under reduced pressure (50°). The residue was dissolved in the MeCN (120 mL) and DCM (120 mL) mixture, then RuO_2_ (0.03 g, 0.2 mmol, 2 mol%) and distilled water (160 mL) were added to the solution. The aqueous layer was acidified with 10% aqueous HCl solution, to pH 8, and then solid KIO_4_ (2.5 g, 0.011 mol, 1.0 equiv.) was added in five portions. The reaction mixture was stirred vigorously at room temperature for 2 days. The progress of the reaction was monitored by TLC in the MeOH/DCM (1/9) mixture. Then isopropyl alcohol (20 mL) was added to quench the reaction and stirring was continued until complete precipitation of RuO_2_ was observed. The reaction mixture was diluted with water (200 mL) and acidified with 10% HCl solution to pH 6. The product was extracted with DCM (3 × 30 mL). The combined organic layers were dried over anhydrous sodium sulfate, and the solvent was evaporated. The residue was filtered through a short pad of silica gel with hexane/AcOEt (1:1) elution. Crude product was purified by crystallization (hexane/AcOEt, 1:1) to give **5b** (4.401 g, 85%) as a white crystalline material.

(20*S*)-3β-*t*-butyldimetylsilyloxy-16-oxo-5α-pregnane-20-carboxylic acid (**5b**): white crystals, m.p. 234–236 °C (hexane/AcOEt). ^1^H NMR (CDCl_3_, 400 MHz) δ 9.64 (bs, 1H), 3.57 (m, 1H), 2.52 (m, 1H), 2.39 (d, *J* = 10.5, 1H), 2.25 (dd, *J*_1_ = 6.9, *J*_2_ = 18.4, 1H), 2.02 (m, 1H), 1.26 (d, *J* = 7.0, 3H), 0.89 (s, 9H), 0.83 (s, 3H), 0.77 (s, 3H), 0.06 (s, 6H). ^13^C NMR (CDCl_3_, 100 MHz) δ, 217.2 (C), 181.9 (C), 72.0 (CH), 65.0 (CH), 54.1 (CH), 50.8 (CH), 44.9 (CH), 42.2 (C), 39.0 (CH_2_), 38.5 (CH_2_), 37.6 (CH), 37.5 (CH_2_), 36.8 (CH_2_), 35.6 (C), 34.4 (CH), 32.1 (CH_2_), 31.8 (CH_2_), 28.4 (CH_2_), 25.9 (3CH_3_), 20.7 (CH_2_), 18.2 (C), 16.4 (CH_3_), 13.1 (CH_3_), 12.3 (CH_3_), −4.6 (2CH_3_). IR (ATR): ν_max_ (cm^−1^): 3300-2500 (broad), 1743, 1721, 1459, 1250, 1097, 1087. ESI-MS 475 [M − H]^−^. HRMS calcd. for C_28_H_47_O_4_Si 475.3249 (M − H)^−^, found 475.3248.

##### Reductive Amination/Cyclization of Oxo-Acid (**5b**) with Ammonium Acetate/Sodium Borohydride

Oxo-acid **5b** (0.15 g, 0.32 mmol, 1 equiv.), NH_4_OAc (0.365 g, 4.74 mmol, 15 equiv.), NaBH_3_CN (0.060 g, 0.91 mmol, 3.0 equiv.) and MeOH (4 mL) were mixed in the 10 mL-tube sealed with a stopper. The resulting suspension was stirred for 40 min at 120 °C under microwave irradiation. Then the reaction mixture was poured to 5% citric acid aqueous solution (15 mL) and product was extracted with DCM (3 × 15 mL). The combined organic layers were washed with brine (1 × 15 mL), dried over anhydrous sodium sulfate and the solvent was evaporated. Purification by silica gel column chromatography gave product **4b** (61% yield) eluted with 1% MeOH/DCM.

##### Procedure for *N*-Alkyl-Lactam **4e**, **4f** - Synthesis from Oxo-Acid **5b**

Oxo-acid **5b** (0.15 g, 0.32 mmol, 1 equiv.), amine (BnNH_2_, *p*-MeOBnNH_2_, 0.91 mmol, 3 equiv.) and THF (3 mL) were added to the 10 mL-tube sealed with a stopper. The resulting suspension was stirred for 20 min at 140 °C under microwave irradiation (a clear yellow solution was formed). Then, NaBH_3_CN (0.060 g, 0.91 mmol, 3.0 equiv.) and MeOH (3 mL) were added to the tube. The solution was heated under the same conditions for another 20 min. Then the reaction mixture was poured into 20 mL of a 5% citric acid aqueous solution and product was extracted with DCM (3 × 20 mL). The combined organic layers were washed with saturated NaCl solution (1 × 20 mL), dried over anhydrous sodium sulfate, and the solvent was evaporated. The crude product was purified by column chromatography on silica gel.

(16*S*,20*S*)-*N*-benzyl-3β-*t*-butyldimetylsilyloxy-5α-pregnane-20,16-carbolactam (**4e**): eluted with hexane/AcOEt/DCM (6:2:2) in 73% yield (0.126 g), as an amorphous solid. ^1^H NMR (CDCl_3_, 400 MHz) δ 7.31 (m, 3H), 7.21 (m, 2H), 5.01 (d, *J* = 14.5, 1H), 3.81 (d, *J* = 14.5, 1H), 3.74 (m, 1H), 3.53 (m, 1H), 2.50 (m, 1H), 1.87 (m, 1H), 1.77 (m, 1H), 1.21 (d, *J* = 7.5, 3H), 0.89 (s, 9H), 0.80 (s, 3H), 0.71 (s, 3H), 0.04 (s, 6H).^13^C NMR (CDCl_3_, 100 MHz) δ, 178.2 (C), 136.6 (C), 128.6 (CH), 128.2 (2CH), 127.4 (CH), 72.0 (CH), 58.8 (CH), 55.6 (CH), 54.8 (CH), 54.5 (CH), 44.9 (CH), 44.3 (CH_2_), 42.0 (C), 38.8 (CH_2_), 38.6 (CH_2_), 37.1 (CH, CH_2_), 35.6 (C), 35.0 (CH), 32.3 (CH_2_), 31.9 (CH_2_), 31.1 (CH_2_), 28.6 (CH_2_), 25.9 (3CH_3_), 20.6 (CH_2_), 18.9 (CH_3_), 18.2 (C), 14.3 (CH_3_), 12.3 (CH_3_), −4.6 (2CH_3_). IR (ATR): ν_max_ (cm^−1^): 1670, 1249, 1090. ESI−MS 550 [M + H]^+^, 572 [M + Na]^+^. HRMS calcd. for C_35_H_56_NO_2_Si 550.4075 (M + H)^+^, found 550.4078.

(16*S*,20*S*)-*N*-(*p*-methoxybenzyl)-3β-*t*-butyldimetylsilyloxy-5α-pregnan-20,16-carbolactam (**4f**): eluted with hexane/AcOEt/DCM (6:2:2) in 75% yield as an amorphous solid. ^1^H NMR (CDCl_3_, 400 MHz) δ 7.14 (d, *J* = 8.6, 2H), 6.85 (d, *J* = 8.6, 2H), 4.95 (d, *J* = 14.4, 1H), 3.80 (s, 3H), 3.75 (d, *J* = 14.4, 1H), 3.72 (m, 1H), 3.54 (m, 1H), 2.48 (q, *J* = 7.4, 1H), 1.86 (m, 1H), 1.20 (d, *J* = 7.4, 3H), 0.89 (s, 9H), 0.80 (s, 3H), 0.70 (s, 3H), 0.05 (s, 6H). ^13^C NMR (CDCl_3_, 100 MHz) δ, 178.1 (C), 158.9 (C), 129.5 (2CH), 128.7 (C), 113.9 (2CH), 72.4 (CH), 58.7 (CH), 55.6 (CH), 55.2 (CH_3_), 54.8 (CH), 54.5 (CH), 44.9 (CH), 43.7 (CH_2_), 42.0 (C), 38.8 (CH_2_), 38.6 (CH_2_), 37.2 (CH, CH_2_), 35.6 (C), 35.0 (CH), 32.3 (CH_2_), 31.9 (CH_2_), 31.1 (CH_2_), 28.6 (CH_2_), 25.9 (3CH_3_), 20.6 (CH_2_), 18.9 (CH_3_), 18.2 (C), 14.3 (CH_3_), 12.4 (CH_3_), −4.6 (2CH_3_),. IR (ATR): ν_max_ (cm^−1^): 1682, 1612, 1512, 1453, 1244, 1090. ESI-MS 580 [M + Na]+, 602 [M + Na]+. HRMS calcd. for C_36_H_58_NO_3_Si 580.4180 (M + H)^+^, found 580.4186.

## 4. Conclusions

To summarize, two alternative procedures for the synthesis of bisnorcholanic lactams, hitherto unknown aza-analogs of naturally occurring vespertilin lactone, were reported. Both elaborated methods provided short and efficient routes to the target lactam as well as to its *N*-alkyl derivatives. A convenient substrate employed in these syntheses was bisnorcholanic lactone, readily available by tigogenin degradation. A key-intermediate in the first strategy was 16-oxo-5α-pregnane-20-carboxyamide obtained in two consecutive reactions: ammonolysis of bisnorcholanic lactone with prepared in situ aminoalane and the 16β-hydroxyl group oxidation. The cyclization of the oxo-amide with a simultaneous deprotection of the 3β-hydroxy group proceeded smoothly under reductive conditions (Et_3_SiH/Bi(TfO)_3_) and provided the desired lactam in a quantitative yield. When the lactone ring-opening was carried out using the aluminum amide reagent prepared from primary amine or its hydrochloride (instead of ammonium chloride), different *N*-alkyl lactams were obtained by employing an analogous protocol. An alternative strategy for the lactam preparation comprised of lactone hydrolysis, oxidation of the obtained in situ hydroxy-acid to oxo-acid, and a microwave-assisted reductive amination. In addition, this procedure can be successfully adapted to the synthesis of *N*-alkylated derivatives of bisnorcholanic lactam by using various amines for the reductive amination step. Of the two elaborated synthetic strategies, the first approach is advantageous as is more efficient and completely stereoselective. This method benefited from the intramolecular reductive amination of the 16-carbonyl group that occurs from the β-side only, contrary to intermolecular reductive amination used in the second protocol, in which the lactam precursor, 16β-amino-5α-pregnanecarboxylic acid, was accompanied by minor amounts of its 16α-amino isomer, which is unable to lactamize due to steric reasons. Further study on the application of bisnorcholanic lactam in the synthesis of steroidal alkaloids and the evaluation of biological activity of different lactam congeners is currently in progress.

## Supplementary Materials

Experimental procedure for synthesis of lactone **1b** and ^1^H-NMR, ^13^C-NMR spectra of compounds **2b**–**5b,** NOE and ROSEY spectra for compound **4a**, respectively.
