# Supplementary material for: A Convenient Synthesis of (16S,20S)-3β-Hydroxy-5α-pregnane-20,16-carbolactam and Its N-alkyl Derivatives
Source: Molecules. 2020 May 20;25(10):2377. doi: 10.3390/molecules25102377 (PMC7287600; doi:10.3390/molecules25102377)
Supplement: Supplementary file 1 [file molecules-25-02377-s001.pdf]

Supplementary Materials for

A convenient synthesis of (16*S*,20*S*)-3β-hydroxy-5*α*-pregnane-20,16-carbolactam and its *N*-alkyl derivatives

Agnieszka Wojtkielewicz*, Damian Pawelski, Przemysław Bazydło, Aneta Baj, Stanisław Witkowski, and Jacek W. Morzycki

Institute of Chemistry, University of Białystok, K. Ciołkowskiego 1K, 15-245 Białystok, Poland; a.wojtkielewicz@uwb.edu.pl (A.W.); d.pawelski@uwb.edu.pl (D.P.); bazydlo_przemyslaw@wp.pl (P.B.) aneta.baj@uwb.edu.pl (A.B.); wit@uwb.edu.pl (S.W.); morzycki@uwb.edu.pl (J.W.M.)

*Correspondence: a.wojtkielewicz@uwb.edu.pl; Tel.: +48-85-738-8043

Table of contents

1. Experimental procedures for synthesis of (16*S*,20*S*)-3β-*t*-butyldimethylsilyloxy-5*α*-pregnane-20,16-carbolactone (**1b**) from tigogenin……………………….…..........S2
2. Spectra (^{1}H NMR, ^{13}C NMR) of compounds **2b** - **5b**…………………………..S3-S32

Experimental procedures for (16S,20S)-3β-*t*-butyldimethylsilyloxy-5α-pregnane-20,16-carbolactone (1b) synthesis from tigogenin

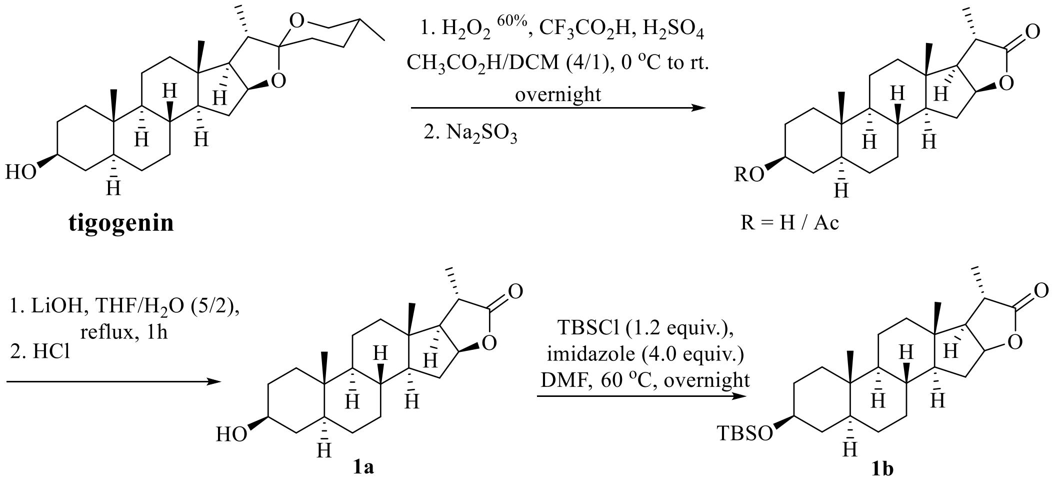

Trifluoroacetic acid (1 mL) and concentrated sulphuric acid (10 mL) were added to the mixture of tigogenin (10 g, 0.024 mol), molecular iodine (0.5 g, 0.002 mol), *n*-Bu_4NBr (0.100 g, 3 x 10^{-4} mol), acetic acid (120 ml) in DCM (30 ml). The multi-component solution was heated at 50° C for 1 hour, then, after cooling to 0°C, 60% aqueous solution of H_2O_2 (20 mL, 0.438 mol, 18.2 equiv.) was slowly added. After 1 h, the ice bath was removed and stirring was continued at room temperature overnight. The progress of the reaction was monitored by TLC. After completion of the reaction, a saturated aqueous Na_2SO_3 solution was carefully added to the mixture (ca. 300 mL). Stirring was continued until the purple colour of the mixture disappeared. After that, the resulting yellow solution was neutralized with 50% aqueous NaOH solution to pH 7 (ca. 120 mL). Product was extracted with DCM (5 x 30 mL). The combined organic layers were concentrated under reduced pressure. Then, LiOH (2.874, 0.120 mol) and a THF/H_2O (5/2) mixture (200 mL) were added to the residue. The resulting suspension was heated at reflux. The progress of saponification was monitored by TLC. After about 1 hour, the solution was acidified with 12 N HCl to pH 3 and stirring was continued for approx.. 10 min at 80°C. After cooling, THF was evaporated under reduced pressure. The residue was diluted with water (200 mL) and product was extracted with AcOEt (5 x 20 mL). The combined organic layers were dried over anhydrous Na_2SO_4, filtered and the solvent was concentrated under reduced pressure. The crude product was purified by crystallization from AcOEt. (16S,20S)-3β-Hydroxy-5α-pregnane-20,16-carbolactone (**1a**) was obtained as a white crystalline material (7.591 g, 91%).

(16*S*,20*S*)-3β-*t*-Butyldimetylsillioxy-5α-pregnane-20,16-carbolactone (**1b**) was obtained in 95% (6.321 g) from (16*S*,20*S*)-3β-hydroxy-23,24-bis-norcholano-22,16-lactone (**1a**, 5 g, 0.014 mol, 1.0 equiv.) according to a standard procedure using imidazole (3.93 g, 0.058 mol, 4 equiv.), TBSCl (2.61 g, 0.017 mol, 1.2 equiv.) in anhydrous DMF (200 mL).

Spectra (^{1}H NMR, ^{13}CNMR) of compounds **2b** - **5b**

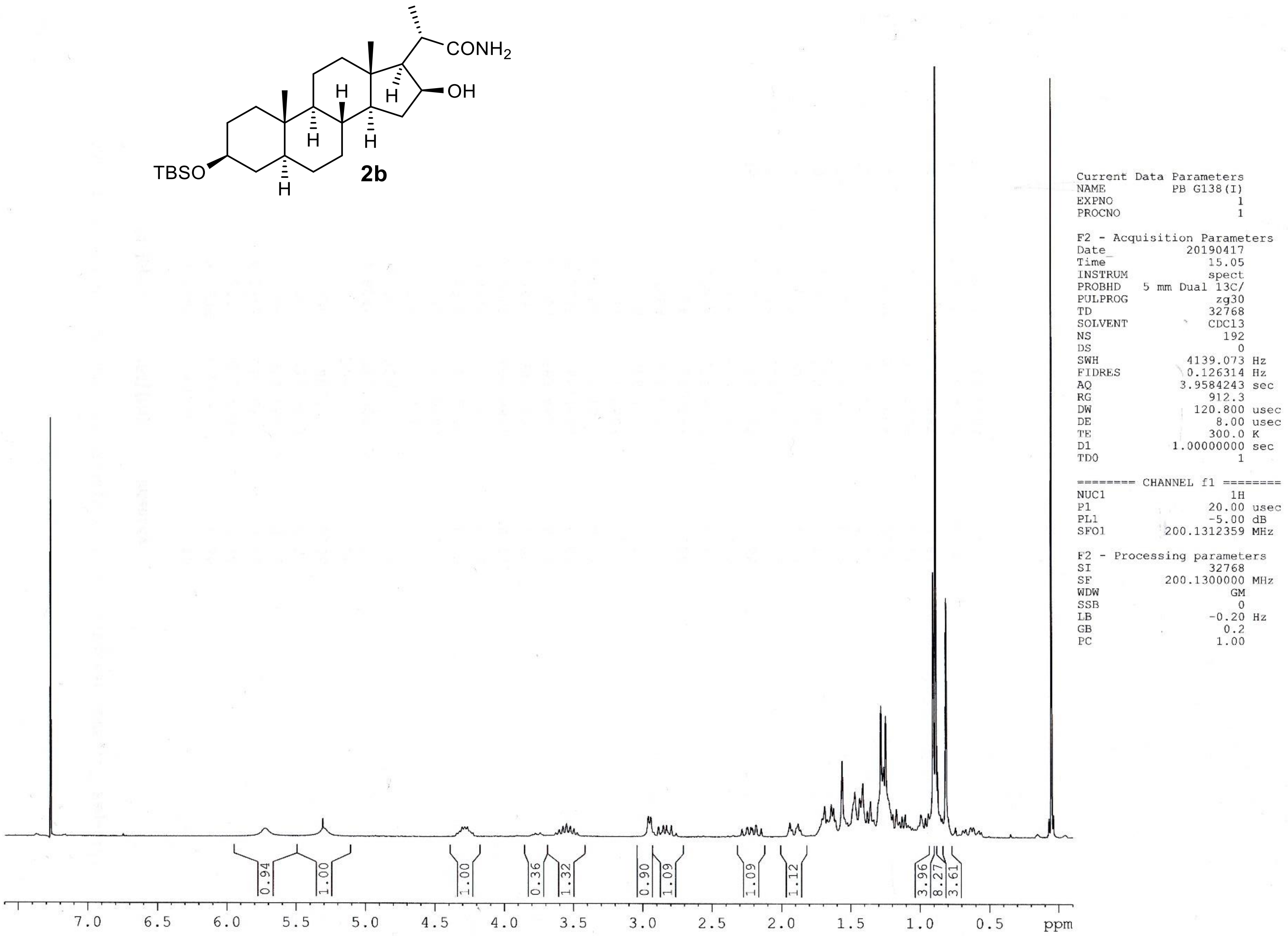
CONH2
OH
H
TBSO
2b
Current Data Parameters
NAME PB G138(I)
EXPNO 1
PROCNO 1
F2 - Acquisition Parameters
Date_ 20190417
Time 15.05
INSTRUM spect
PROBHD 5 mm Dual 13C/
PULPROG zg30
TD 32768
SOLVENT CDCl3
NS 192
DS 0
SWH 4139.073 Hz
FIDRES 0.126314 Hz
AQ 3.9584243 sec
RG 912.3
DW 120.800 usec
DE 8.00 usec
TE 300.0 K
D1 1.00000000 sec
TD0 1
======== CHANNEL f1 ========
NUC1 1H
P1 20.00 usec
PL1 -5.00 dB
SFO1 200.1312359 MHz
F2 - Processing parameters
SI 32768
SF 200.1300000 MHz
WDW GM
SSB 0
LB -0.20 Hz
GB 0.2
PC 1.00
0.94
1.00
1.00
0.36
1.32
0.90
1.09
1.09
1.12
3.96
8.27
3.61
7.0
6.5
6.0
5.5
5.0
4.5
4.0
3.5
3.0
2.5
2.0
1.5
1.0
0.5
ppm

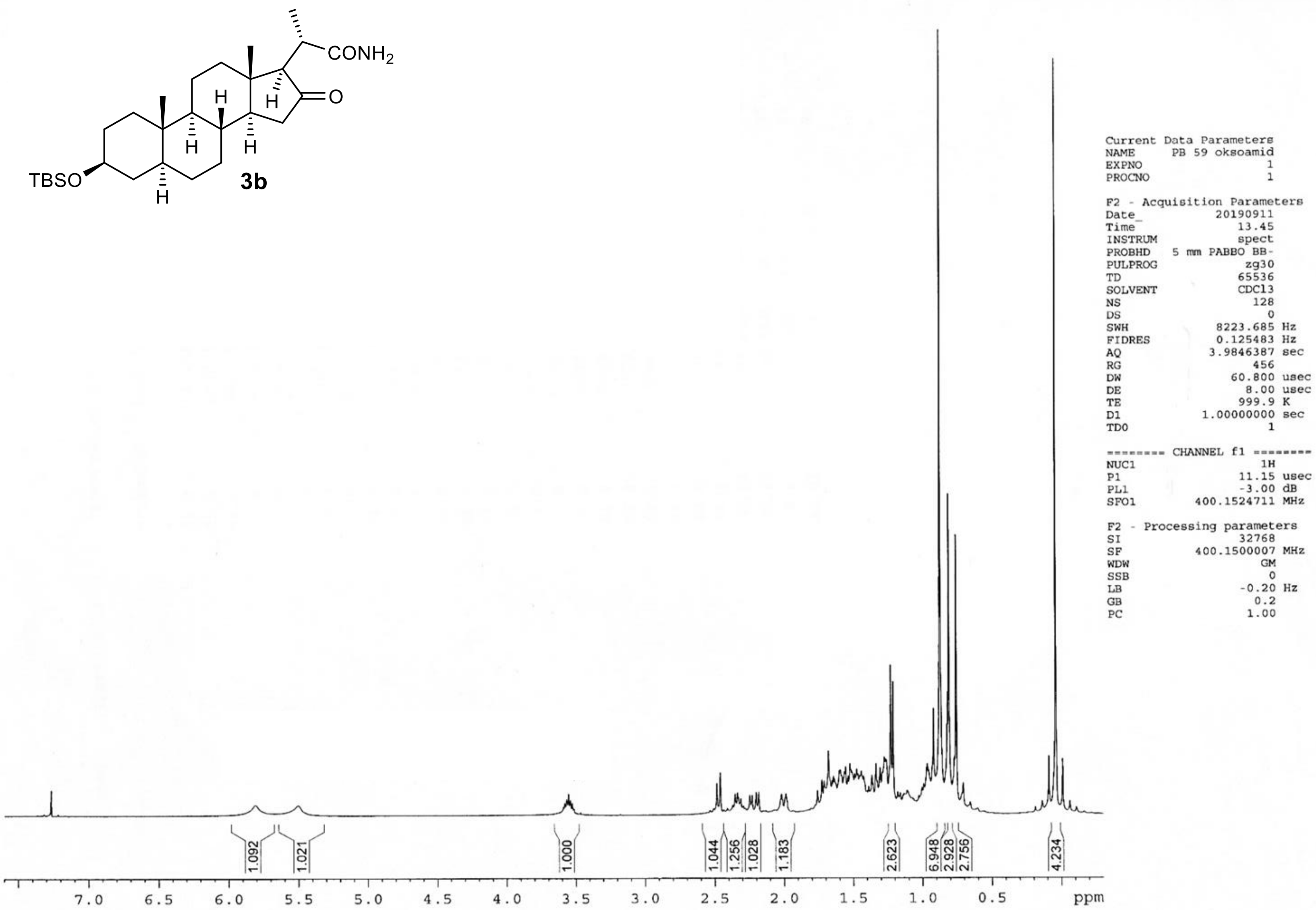
CONH2
O
H
H
H
H
TBSO
H
3b
Current Data Parameters
NAME PB 59 oksoamid
EXPNO 1
PROCNO 1
F2 - Acquisition Parameters
Date_ 20190911
Time_ 13.45
INSTRUM spect
PROBHD 5 mm PABBO BB-
PULPROG zg30
TD 65536
SOLVENT CDCl3
NS 128
DS 0
SWH 8223.685 Hz
FIDRES 0.125483 Hz
AQ 3.9846387 sec
RG 456
DW 60.800 usec
DE 8.00 usec
TE 999.9 K
D1 1.00000000 sec
TD0 1
======== CHANNEL f1 ========
NUC1 1H
P1 11.15 usec
PL1 -3.00 dB
SFO1 400.1524711 MHz
F2 - Processing parameters
SI 32768
SF 400.1500007 MHz
WDW GM
SSB 0
LB -0.20 Hz
GB 0.2
PC 1.00
1.092
1.021
1.000
1.044
1.256
1.028
1.183
2.623
6.948
2.928
2.756
4.234
7.0
6.5
6.0
5.5
5.0
4.5
4.0
3.5
3.0
2.5
2.0
1.5
1.0
0.5
ppm

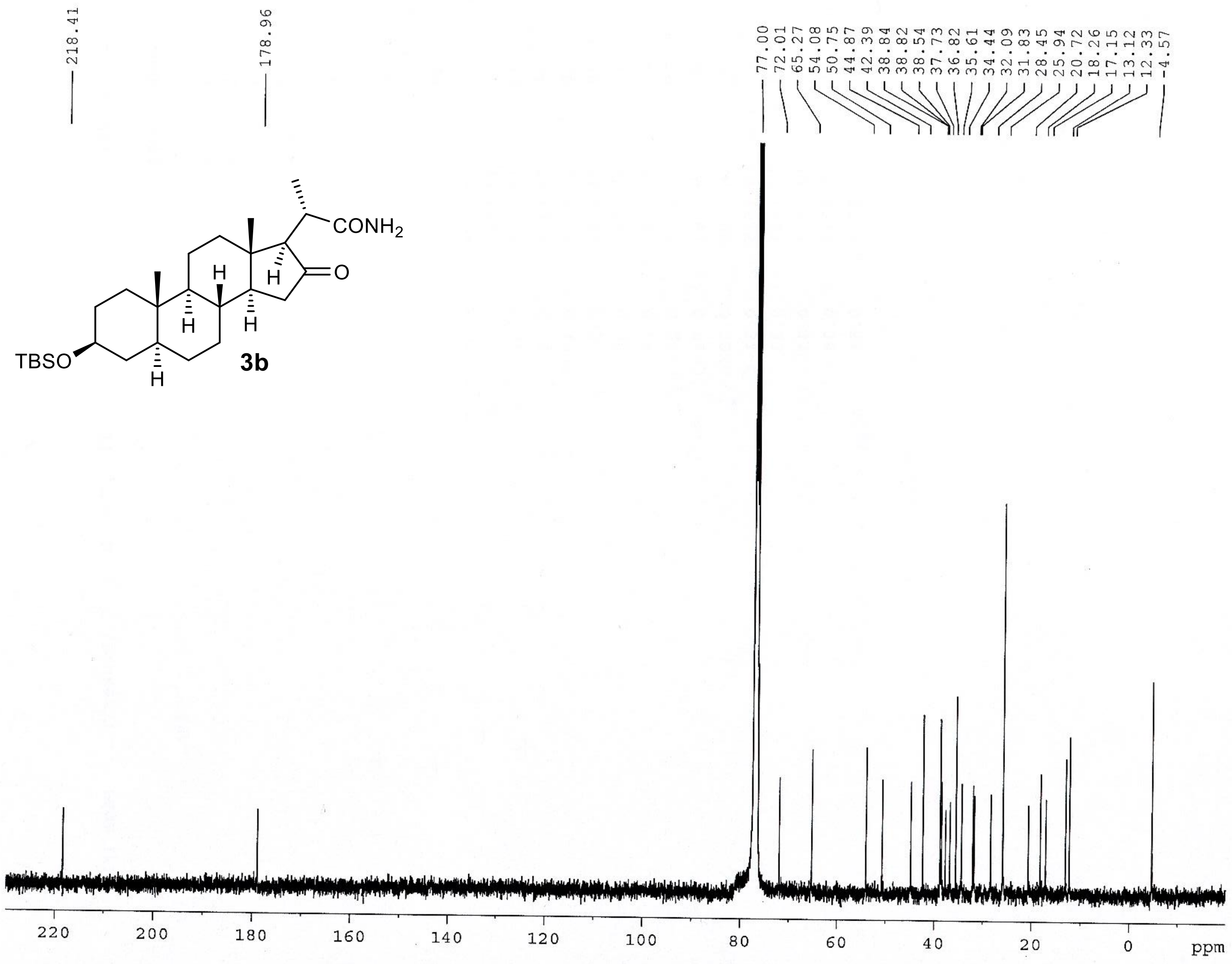

Current Data Parameters
NAME AW G328II'IICC
EXPNO 2
PROCNO 1

F2 - Acquisition Parameters
Date_ 20190603
Time 8.58
INSTRUM spect
PROBHD 5 mm PABBO BB-
PULPROG zgpg30
TD 65536
SOLVENT CDCl3
NS 42000
DS 4
SWH 32051.281 Hz
FIDRES 0.489064 Hz
AQ 1.0224116 sec
RG 2050
DW 15.600 usec
DE 6.00 usec
TE 999.9 K
D1 2.00000000 sec
d11 0.03000000 sec
DELTA 1.89999998 sec
TD0 1

======== CHANNEL f1 ========
NUC1 13C
P1 27.50 usec
PL1 -1.00 dB
SFO1 100.6298721 MHz

======== CHANNEL f2 ========
CPDPRG2 waltz16
NUC2 1H
PCPD2 100.00 usec
PL2 -3.00 dB
PL12 13.65 dB
PL13 18.00 dB
SFO2 400.1516006 MHz

F2 - Processing parameters
SI 32768
SF 100.6177984 MHz
WDW EM
SSB 0
LB 1.00 Hz
GB 0
PC 0.20

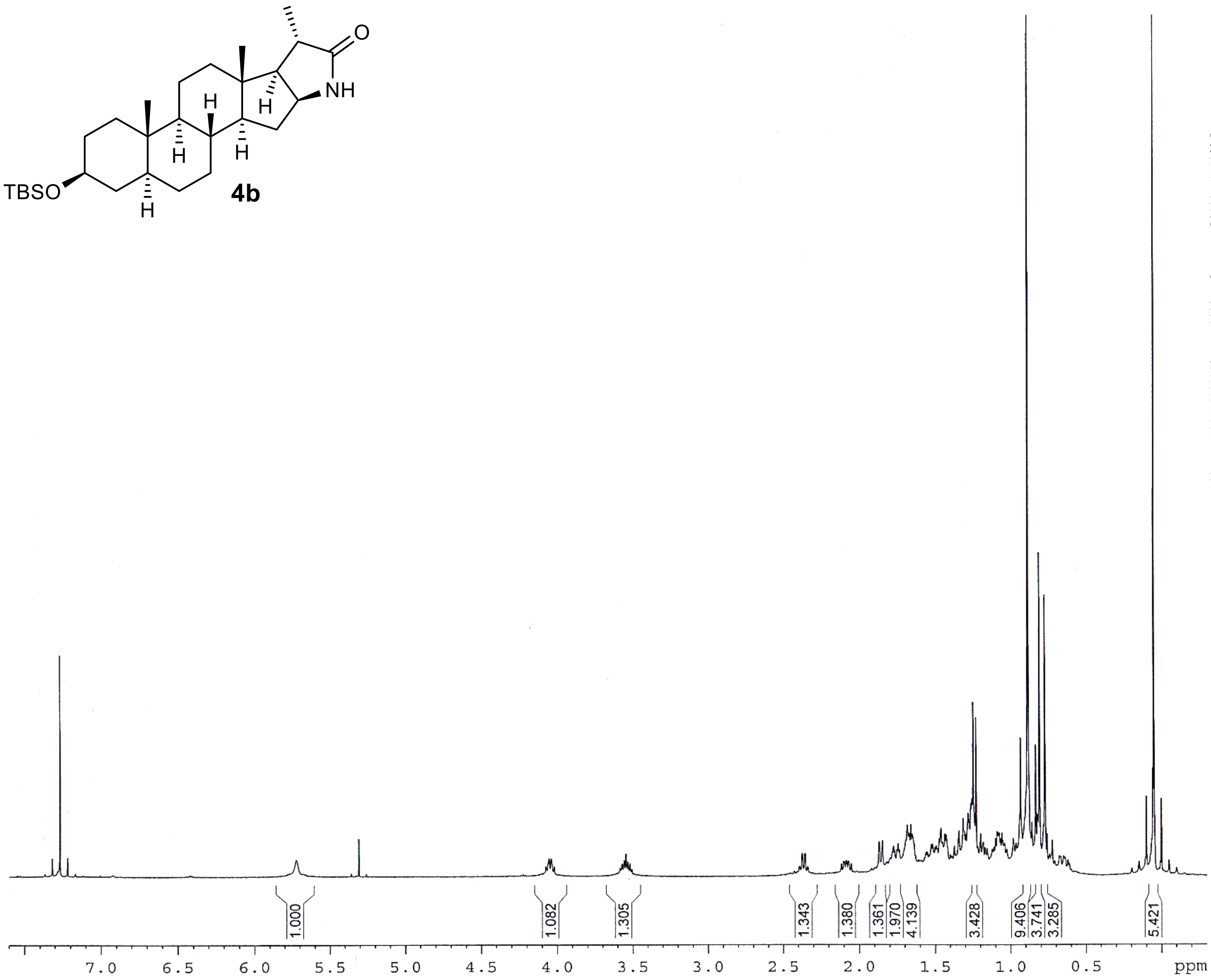

Current Data Parameters
NAME PB 63 Laktam cz F4-8
EXPNO 1
PROCNO 1

F2 - Acquisition Parameters
Date_ 20190927
Time 12.43
INSTRUM spect
PROBHD 5 mm PABBO BB-
PULPROG zg30
TD 65536
SOLVENT CDCl3
NS 192
DS 0
SWH 8223.685 Hz
FIDRES 0.125483 Hz
AQ 3.9846387 sec
RG 203
DW 60.800 usec
DE 8.00 usec
TE 999.9 K
D1 1.00000000 sec
TD0 1

======== CHANNEL f1 ========
NUC1 1H
P1 25.00 usec
PL1 -3.00 dB
SFO1 400.1524711 MHz

F2 - Processing parameters
SI 32768
SF 400.1500007 MHz
WDW GM
SSB 0
LB -0.20 Hz
GB 0.2
PC 1.00

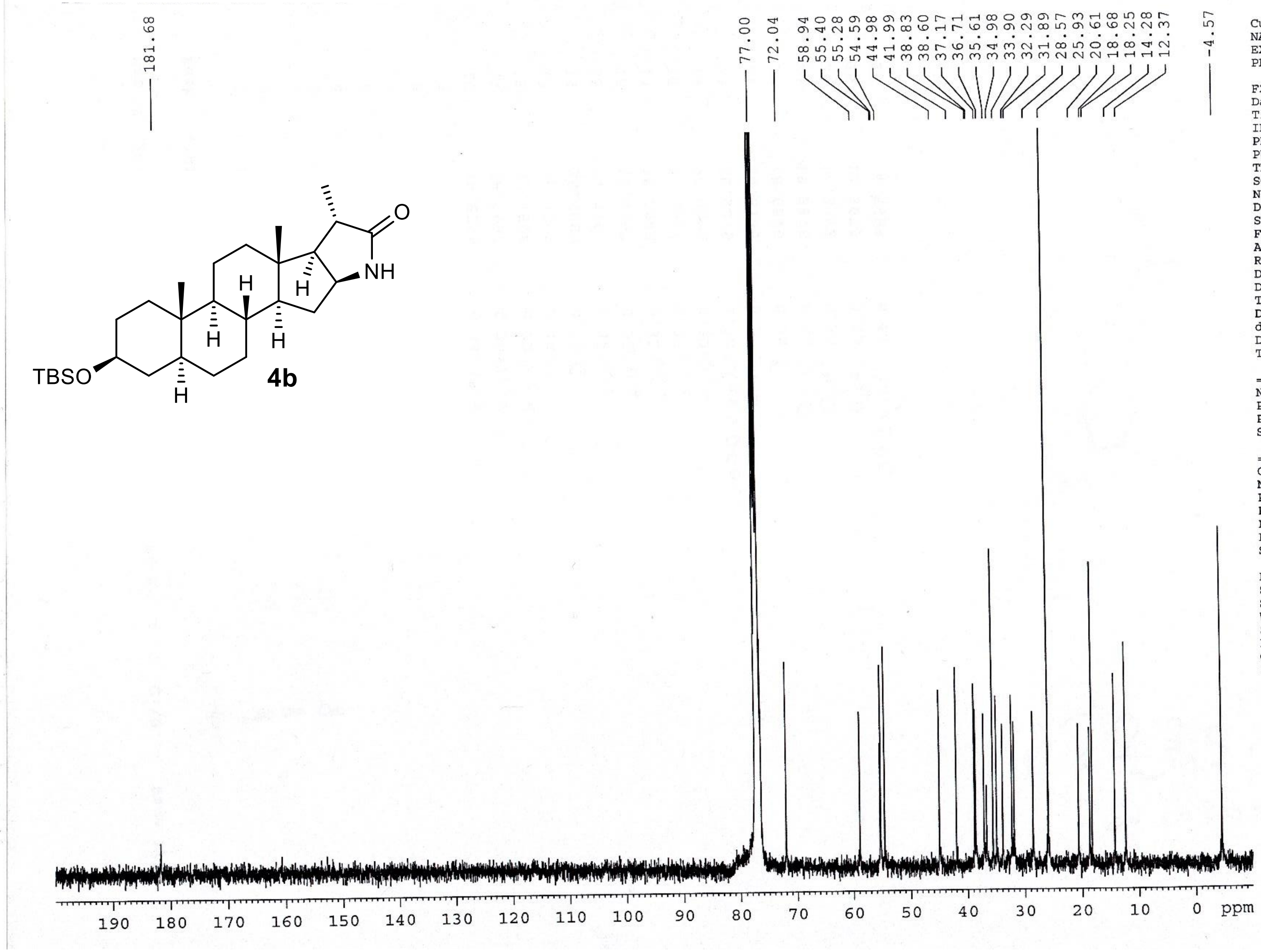

Current Data Parameters
NAME PB 63 Laktam cz F4-8
EXPNO 2
PROCNO 1

F2 - Acquisition Parameters
Date_ 20190929
Time 20.53
INSTRUM spect
PROBHD 5 mm PABBO BB-
PULPROG zgpg30
TD 65536
SOLVENT CDCl3
NS 30720
DS 4
SWH 32051.281 Hz
FIDRES 0.489064 Hz
AQ 1.0224116 sec
RG 287
DW 15.600 usec
DE 6.00 usec
TE 999.9 K
D1 2.00000000 sec
d11 0.03000000 sec
DELTA 1.89999998 sec
TD0 1

======== CHANNEL f1 ========
NUC1 13C
P1 27.50 usec
PL1 -1.00 dB
SFO1 100.6298721 MHz

======== CHANNEL f2 ========
CPDPRG2 waltz16
NUC2 1H
PCPD2 100.00 usec
PL2 -3.00 dB
PL12 13.65 dB
PL13 18.00 dB
SFO2 400.1516006 MHz

F2 - Processing parameters
SI 32768
SF 100.6177991 MHz
WDW EM
SSB 0
LB 1.00 Hz
GB 0
PC 0.20

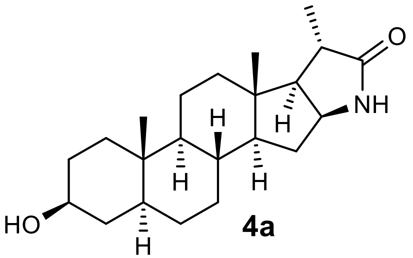

4a

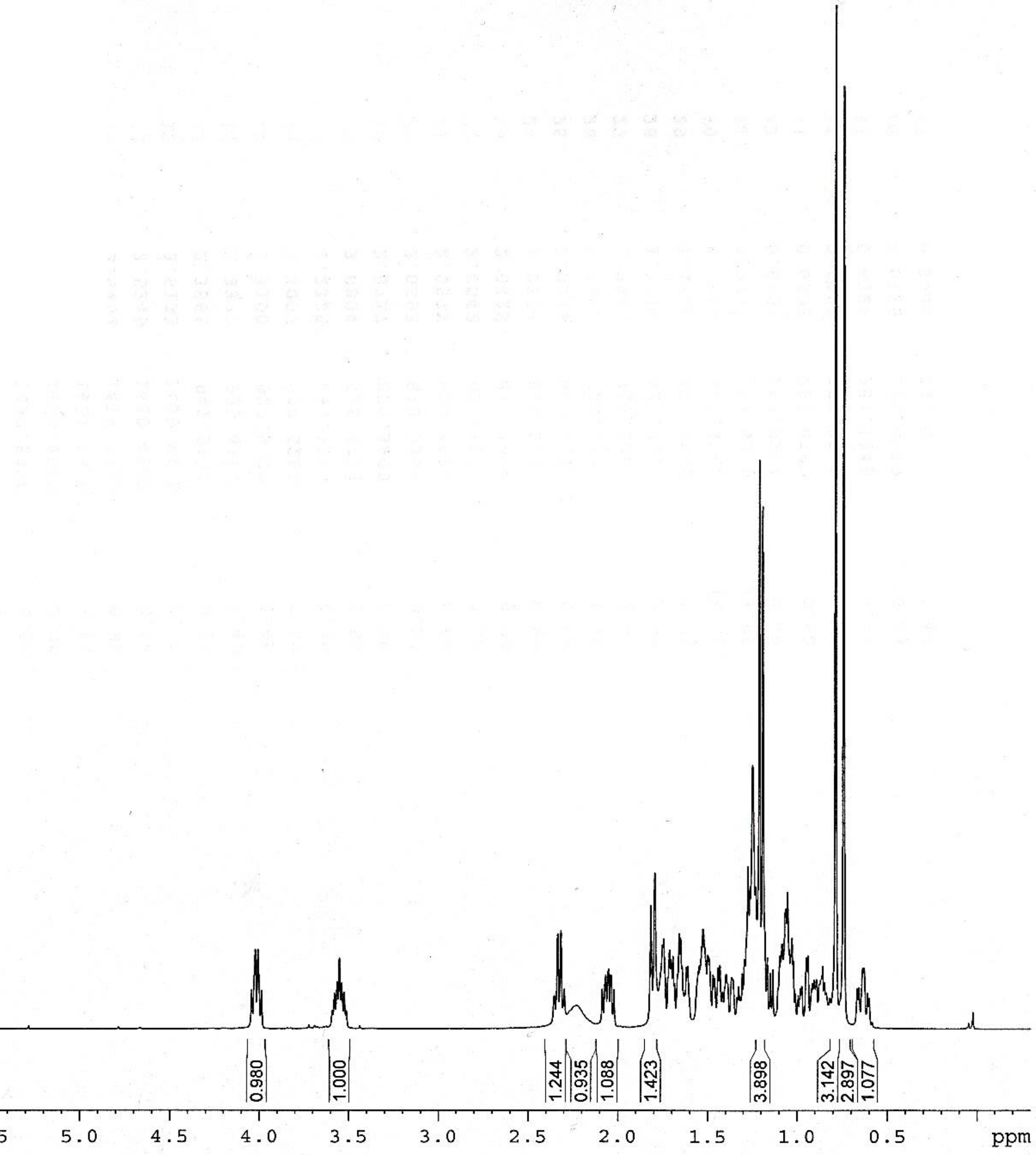


```
Current Data Parameters
NAME        PB LAKTAM-OH'
EXPNO                  11
PROCNO                  1

F2 - Acquisition Parameters
Date_            20200310
Time                13.05
INSTRUM             spect
PROBHD     5 mm PABBO BB-
PULPROG              zg30
TD                  65536
SOLVENT             CDCl3
NS                     64
DS                      0
SWH              8223.685 Hz
FIDRES           0.125483 Hz
AQ              3.9846387 sec
RG                   90.5
DW                 60.800 usec
DE                   8.00 usec
TE                  296.6 K
D1             1.00000000 sec
TD0                     1

======== CHANNEL f1 ========
NUC1                   1H
P1                  11.15 usec
PL1                 -3.00 dB
SFO1          400.1524711 MHz

F2 - Processing parameters
SI                  32768
SF            400.1500007 MHz
WDW                    GM
SSB                     0
LB                  -0.20 Hz
GB                    0.2
PC                   1.00
```

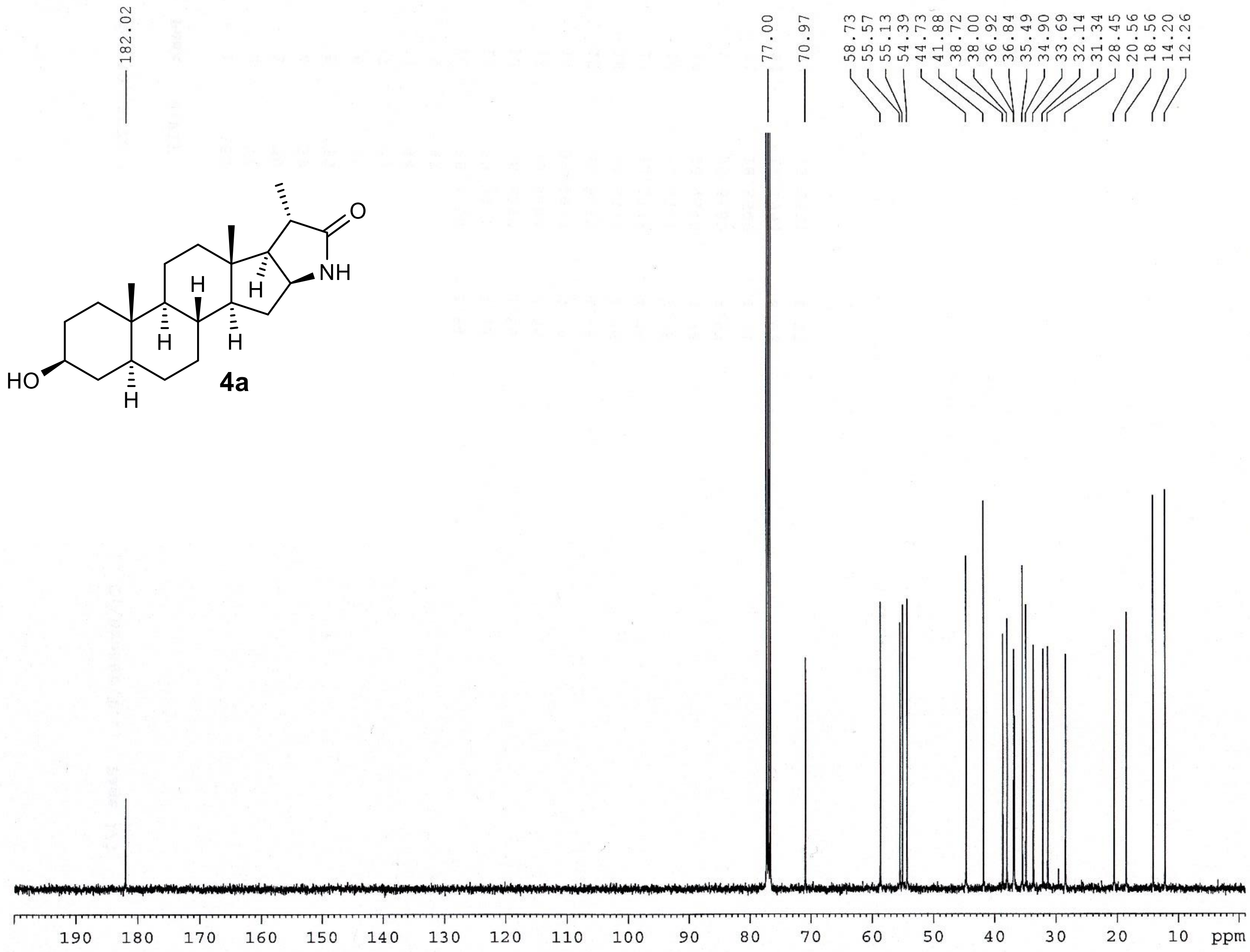


```
Current Data Parameters
NAME       PB LAKTAM-OH'
EXPNO                  2
PROCNO                 1

F2 - Acquisition Parameters
Date_           20200310
Time               13.14
INSTRUM            spect
PROBHD   5 mm PABBO BB-
PULPROG          zgpg30
TD                 65536
SOLVENT            CDCl3
NS                   768
DS                     4
SWH          32051.281 Hz
FIDRES        0.489064 Hz
AQ           1.0224116 sec
RG                  2050
DW              15.600 usec
DE                6.00 usec
TE               297.0 K
D1          2.00000000 sec
d11         0.03000000 sec
DELTA       1.89999998 sec
TD0                    1

======== CHANNEL f1 ========
NUC1                 13C
P1               27.50 usec
PL1              -1.00 dB
SFO1      100.6298721 MHz

======== CHANNEL f2 ========
CPDPRG2          waltz16
NUC2                  1H
PCPD2           100.00 usec
PL2              -3.00 dB
PL12             13.65 dB
PL13             18.00 dB
SFO2      400.1516006 MHz

F2 - Processing parameters
SI                 32768
SF        100.6178053 MHz
WDW                   EM
SSB                    0
LB                1.00 Hz
GB                     0
PC                  0.20
```

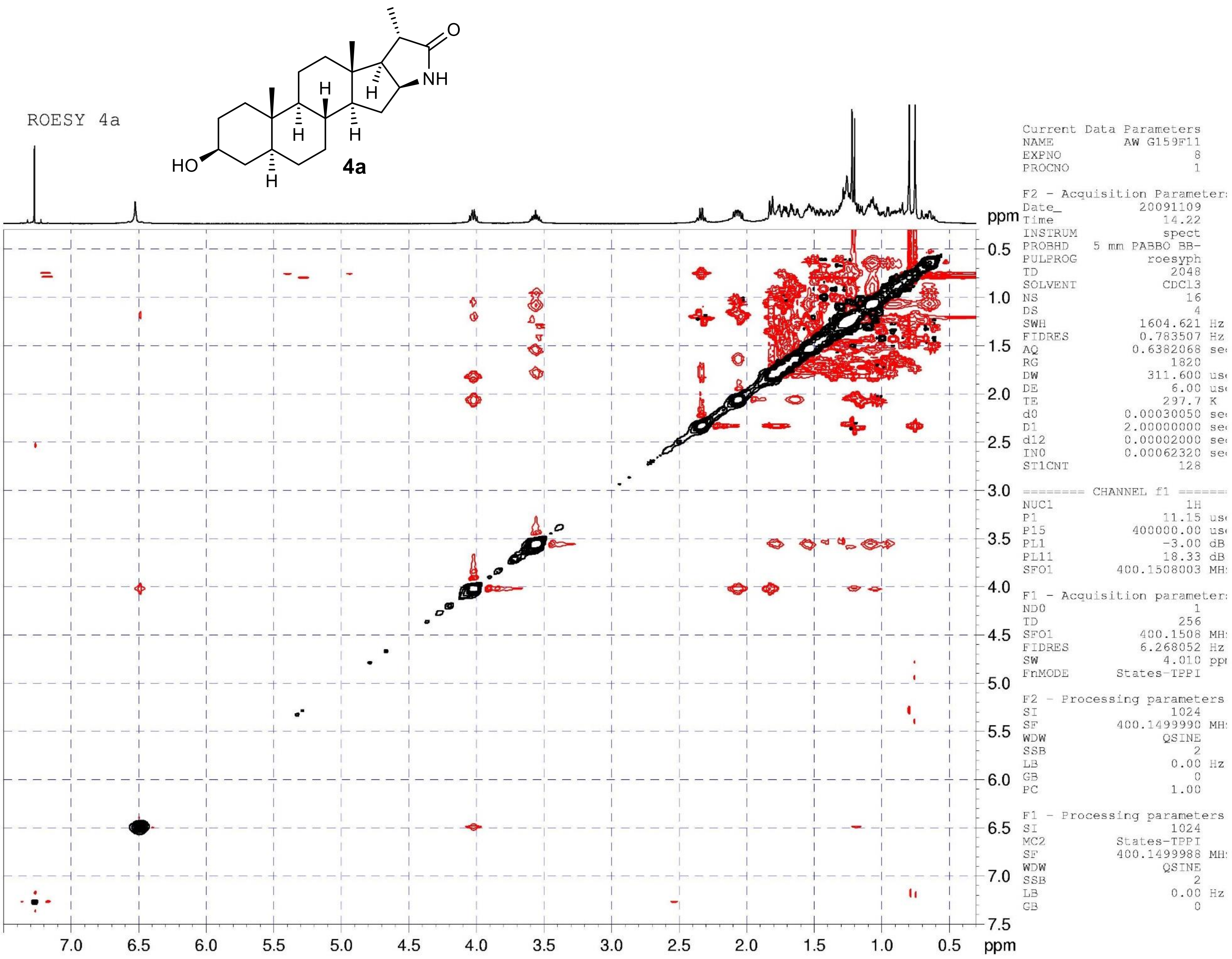
ROESY 4a
HO
H
NH
O
4a
Current Data Parameters
NAME AW G159F11
EXPNO 8
PROCNO 1
F2 - Acquisition Parameters
Date_ 20091109
Time 14.22
INSTRUM spect
PROBHD 5 mm PABBO BB-
PULPROG roesyph
TD 2048
SOLVENT CDCl3
NS 16
DS 4
SWH 1604.621 Hz
FIDRES 0.783507 Hz
AQ 0.6382068 se
RG 1820
DW 311.600 us
DE 6.00 us
TE 297.7 K
d0 0.00030050 se
D1 2.00000000 se
d12 0.00002000 se
IN0 0.00062320 se
ST1CNT 128
======== CHANNEL f1 ======
NUC1 1H
P1 11.15 us
P15 400000.00 us
PL1 -3.00 dB
PL11 18.33 dB
SFO1 400.1508003 MH
F1 - Acquisition parameters
ND0 1
TD 256
SFO1 400.1508 MH
FIDRES 6.268052 Hz
SW 4.010 pp
FnMODE States-TPPI
F2 - Processing parameters
SI 1024
SF 400.1499990 MH
WDW QSINE
SSB 2
LB 0.00 Hz
GB 0
PC 1.00
F1 - Processing parameters
SI 1024
MC2 States-TPPI
SF 400.1499988 MH
WDW QSINE
SSB 2
LB 0.00 Hz
GB 0
ppm
7.0 6.5 6.0 5.5 5.0 4.5 4.0 3.5 3.0 2.5 2.0 1.5 1.0 0.5

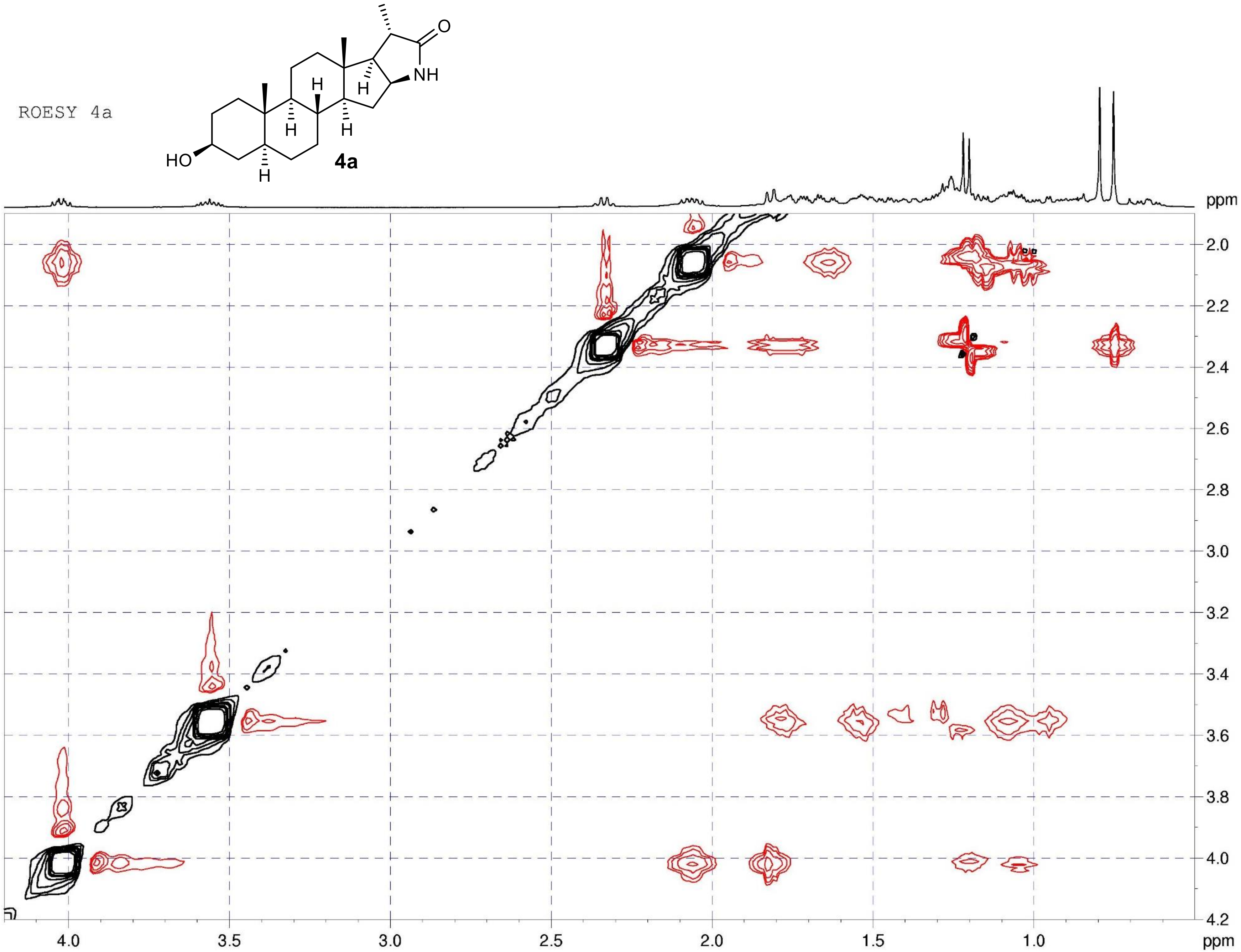
ROESY 4a
HO
NH
O
H
4a
ppm
4.0
3.5
3.0
2.5
2.0
1.5
1.0
2.2
2.4
2.6
2.8
3.2
3.4
3.6
3.8
4.2

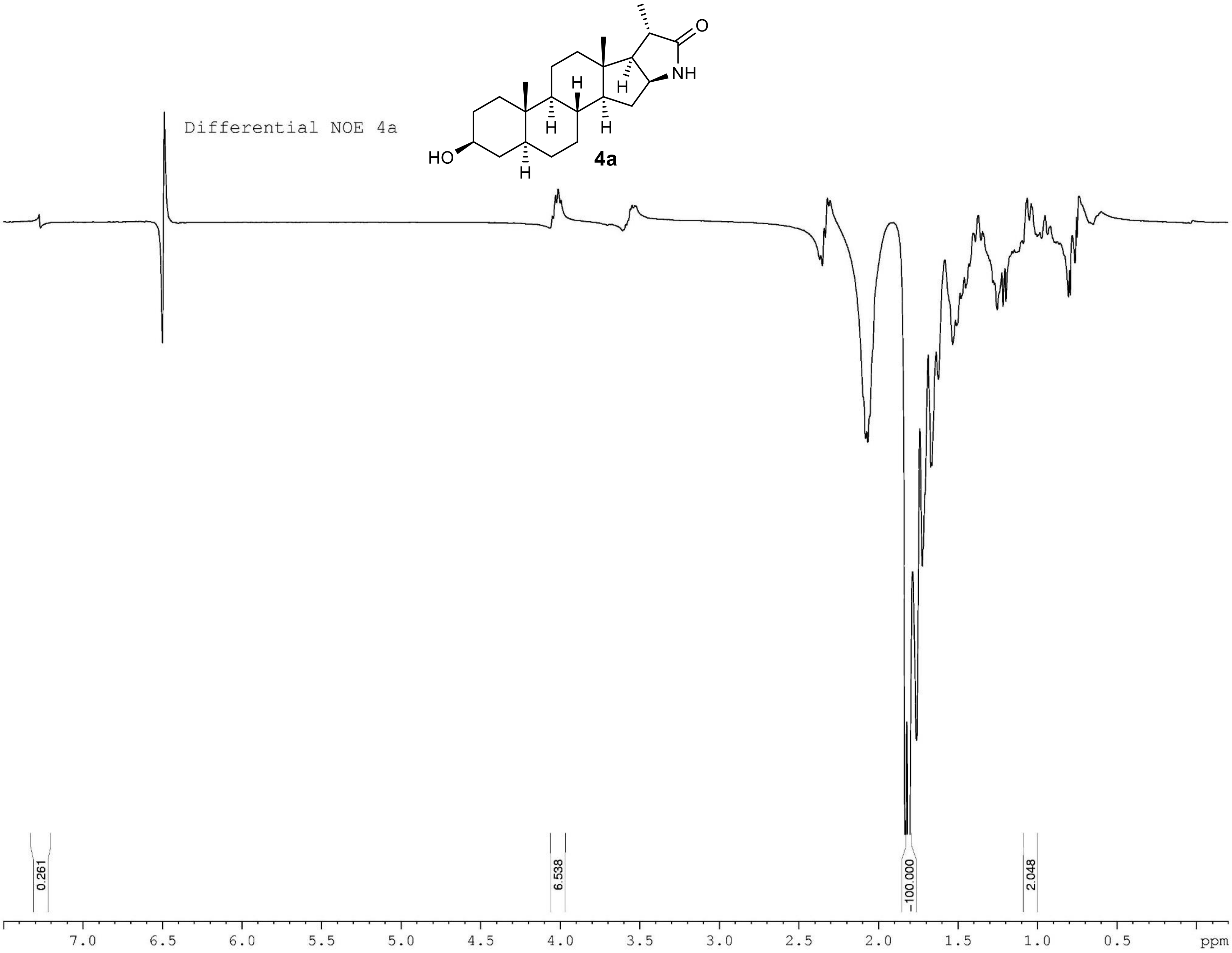
Differential NOE 4a
HO
NH
O
H
4a
0.261
6.538
-100.000
2.048
7.0
6.5
6.0
5.5
5.0
4.5
4.0
3.5
3.0
2.5
2.0
1.5
1.0
0.5
ppm

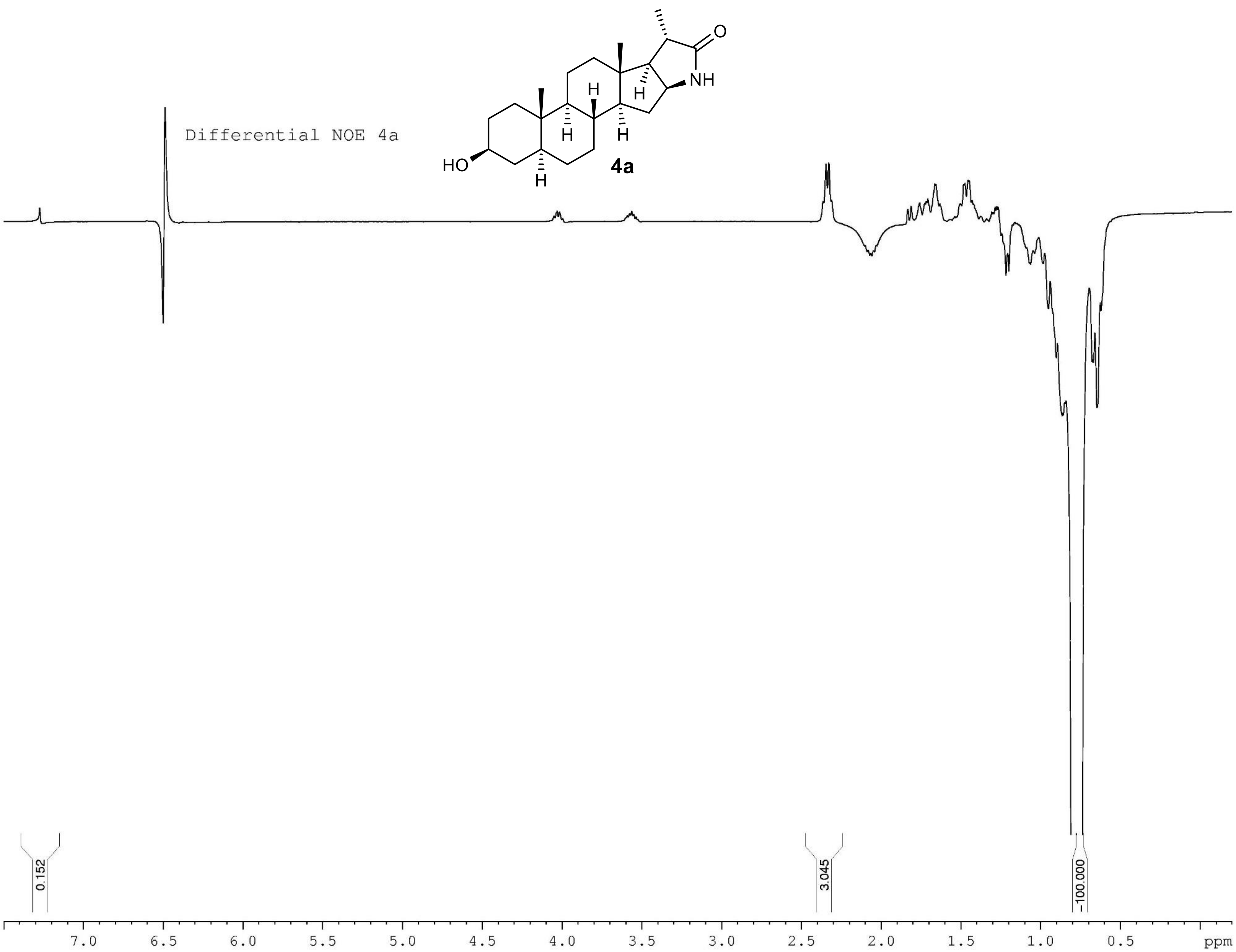
Differential NOE 4a
HO
H
NH
O
4a
0.152
3.045
-100.000
7.0
6.5
6.0
5.5
5.0
4.5
4.0
3.5
3.0
2.5
2.0
1.5
1.0
0.5
ppm

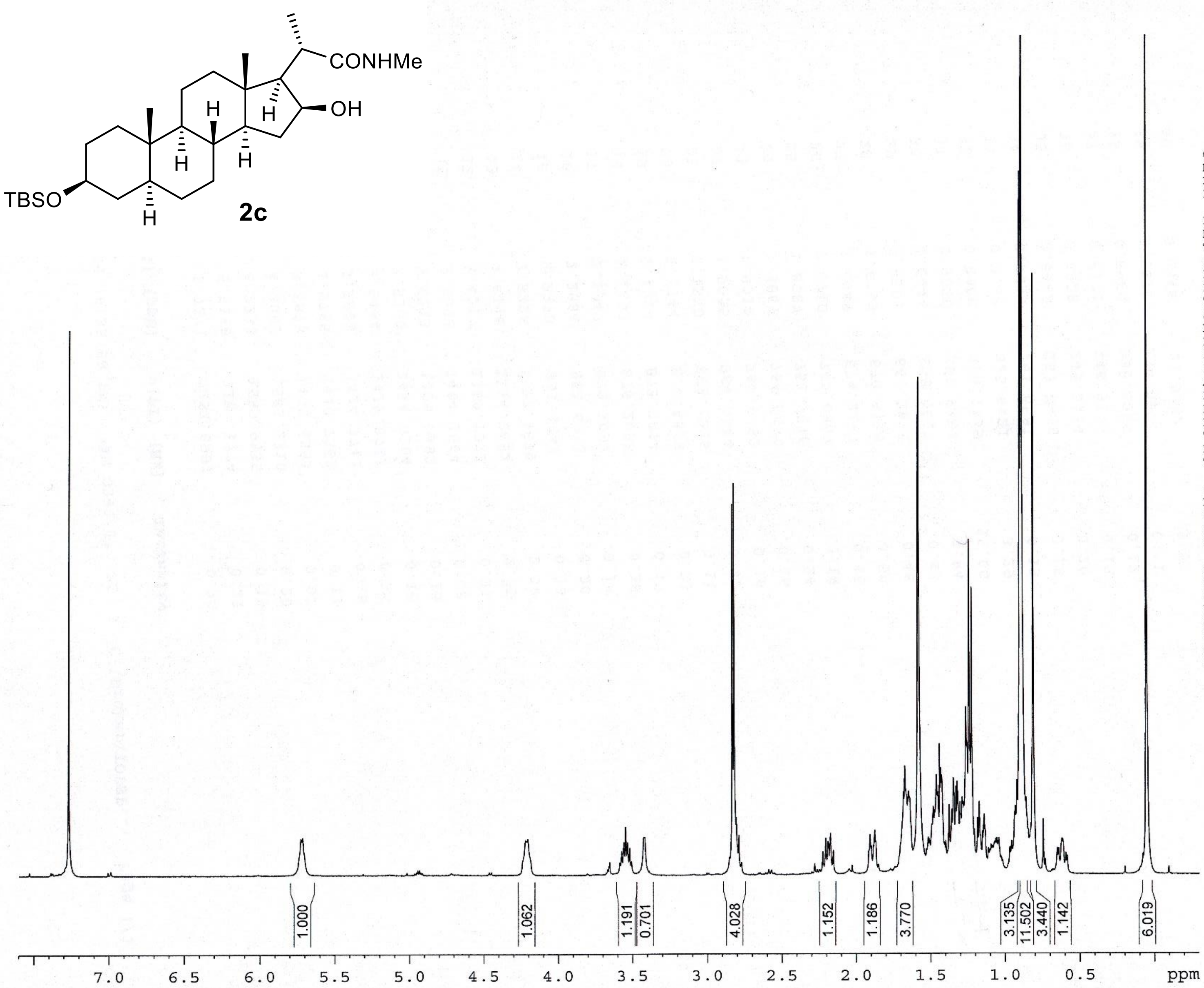


```
Current Data Parameters
NAME             AW G341 I
EXPNO                   11
PROCNO                   1

F2 - Acquisition Parameters
Date_             20200319
Time                 15.38
INSTRUM              spect
PROBHD   5 mm PABBO BB-
PULPROG               zg30
TD                   65536
SOLVENT              CDCl3
NS                     128
DS                       0
SWH             8223.685 Hz
FIDRES          0.125483 Hz
AQ             3.9846387 sec
RG                      32
DW                60.800 usec
DE                  8.00 usec
TE                 999.9 K
D1            1.00000000 sec
TD0                      1

======== CHANNEL f1 ========
NUC1                    1H
P1                 64.00 usec
PL1                -3.00 dB
SFO1       400.1524711 MHz

F2 - Processing parameters
SI                   32768
SF          400.1500000 MHz
WDW                     GM
SSB                      0
LB                   -0.20 Hz
GB                     0.2
PC                    1.00
```

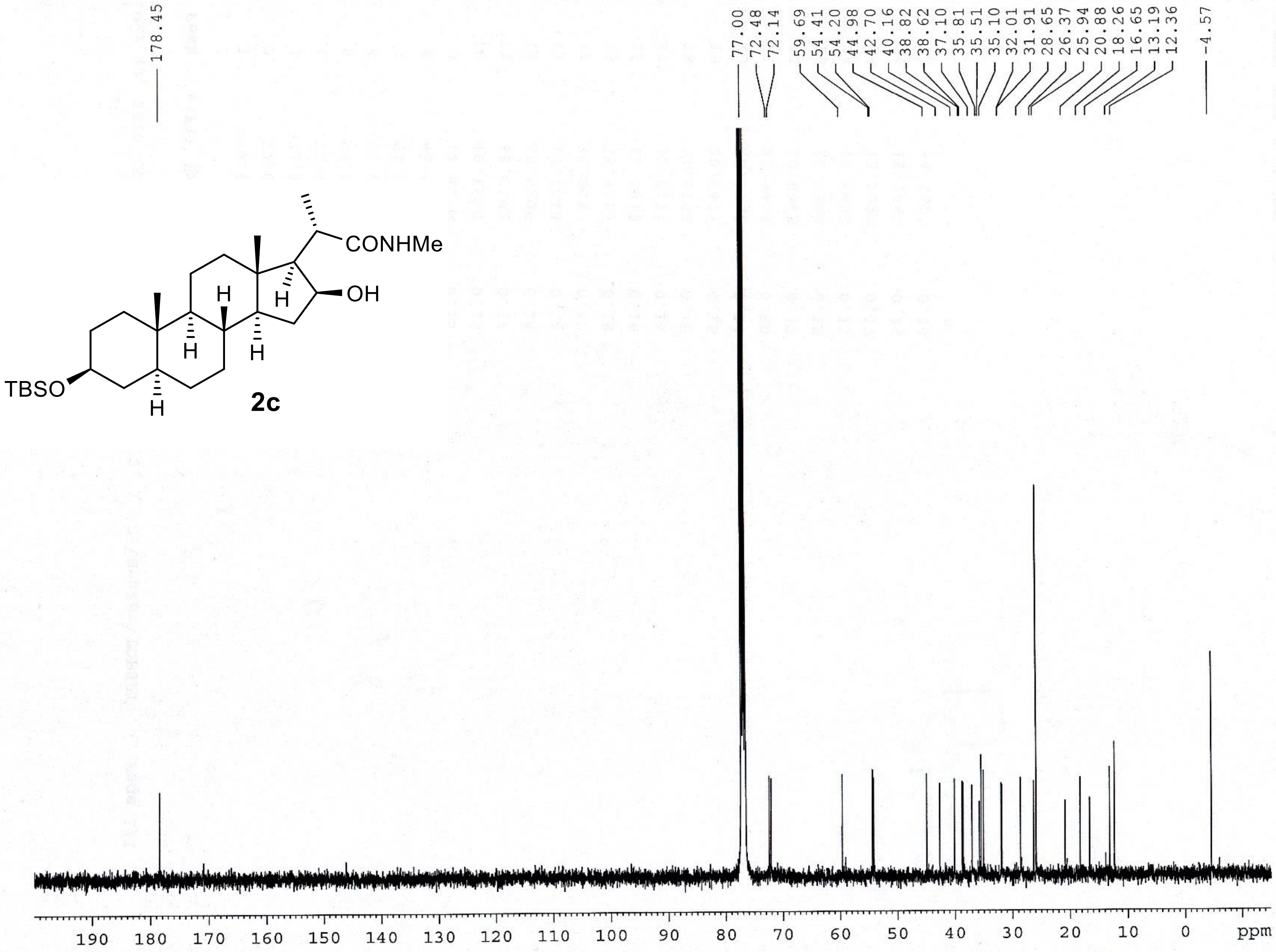

Current Data Parameters
NAME AW G341 I
EXPNO 2
PROCNO 1

F2 - Acquisition Parameters
Date_ 20200320
Time 8.40
INSTRUM spect
PROBHD 5 mm PABBO BB-
PULPROG zgpg30
TD 65536
SOLVENT CDCl3
NS 16384
DS 4
SWH 32051.281 Hz
FIDRES 0.489064 Hz
AQ 1.0224116 sec
RG 128
DW 15.600 usec
DE 6.00 usec
TE 999.9 K
D1 2.00000000 sec
d11 0.03000000 sec
DELTA 1.89999998 sec
TD0 1

======== CHANNEL f1 ========
NUC1 13C
P1 27.50 usec
PL1 -1.00 dB
SFO1 100.6298721 MHz

======== CHANNEL f2 ========
CPDPRG2 waltz16
NUC2 1H
PCPD2 100.00 usec
PL2 -3.00 dB
PL12 13.65 dB
PL13 18.00 dB
SFO2 400.1516006 MHz

F2 - Processing parameters
SI 32768
SF 100.6177982 MHz
WDW EM
SSB 0
LB 1.00 Hz
GB 0
PC 0.20

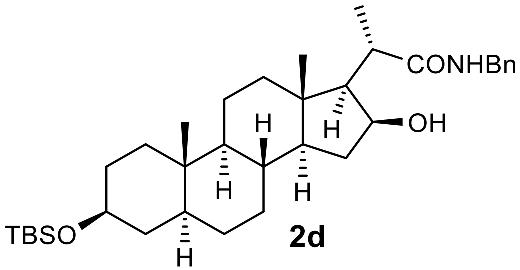
CONHBn
OH
H
H
H
H
H
TBSO

2d

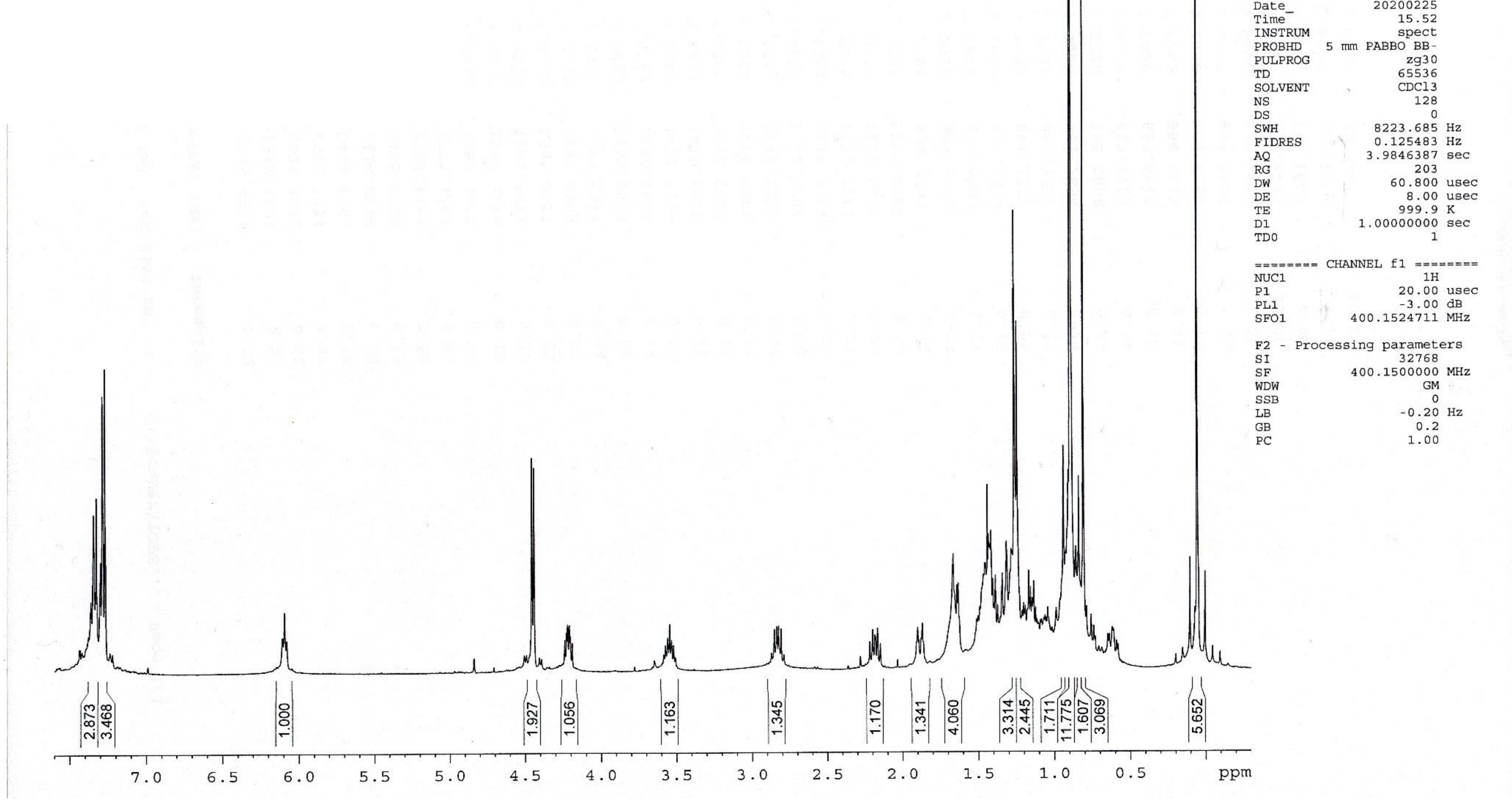
Current Data Parameters
NAME AW G340 sm
EXPNO 1
PROCNO 1
F2 - Acquisition Parameters
Date_ 20200225
Time 15.52
INSTRUM spect
PROBHD 5 mm PABBO BB-
PULPROG zg30
TD 65536
SOLVENT CDCl3
NS 128
DS 0
SWH 8223.685 Hz
FIDRES 0.125483 Hz
AQ 3.9846387 sec
RG 203
DW 60.800 usec
DE 8.00 usec
TE 999.9 K
D1 1.00000000 sec
TD0 1
======== CHANNEL f1 ========
NUC1 1H
P1 20.00 usec
PL1 -3.00 dB
SFO1 400.1524711 MHz
F2 - Processing parameters
SI 32768
SF 400.1500000 MHz
WDW GM
SSB 0
LB -0.20 Hz
GB 0.2
PC 1.00
2.873
3.468
1.000
1.927
1.056
1.163
1.345
1.170
1.341
4.060
3.314
2.445
1.711
11.775
1.607
3.069
5.652
7.0
6.5
6.0
5.5
5.0
4.5
4.0
3.5
3.0
2.5
2.0
1.5
1.0
0.5
ppm

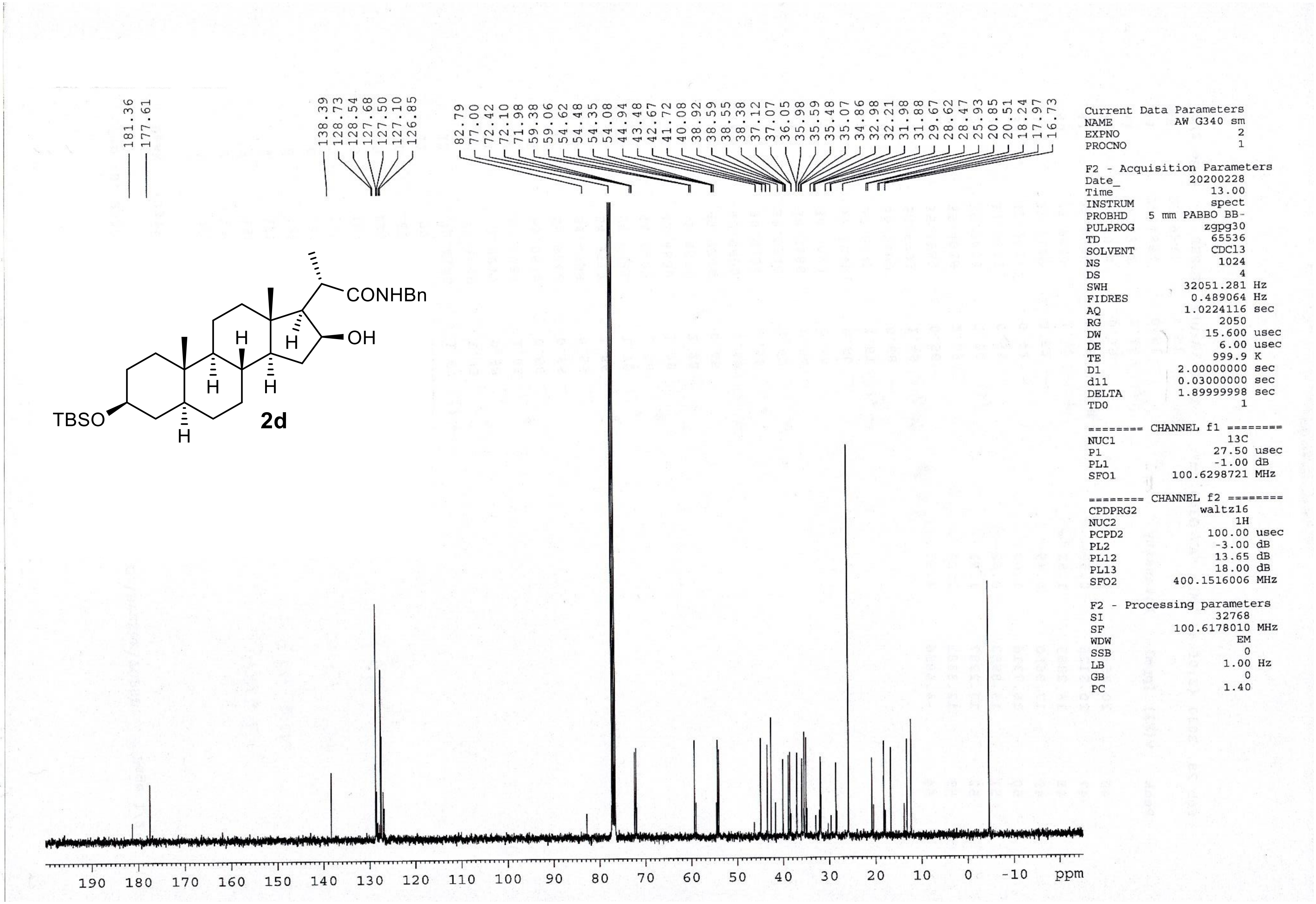

181.36
177.61
138.39
128.73
128.54
127.68
127.50
127.10
126.85
82.79
77.00
72.42
72.10
71.98
59.38
59.06
54.62
54.48
54.35
54.08
44.94
43.48
42.67
41.72
40.08
38.92
38.59
38.55
38.38
37.12
37.07
36.05
35.98
35.59
35.48
35.07
34.86
32.98
32.21
31.98
31.88
29.67
28.62
28.47
25.93
20.85
20.51
18.24
17.97
16.73
CONHBn
OH
H
TBSO
2d
Current Data Parameters
NAME AW G340 sm
EXPNO 2
PROCNO 1
F2 - Acquisition Parameters
Date_ 20200228
Time 13.00
INSTRUM spect
PROBHD 5 mm PABBO BB-
PULPROG zgpg30
TD 65536
SOLVENT CDCl3
NS 1024
DS 4
SWH 32051.281 Hz
FIDRES 0.489064 Hz
AQ 1.0224116 sec
RG 2050
DW 15.600 usec
DE 6.00 usec
TE 999.9 K
D1 2.00000000 sec
d11 0.03000000 sec
DELTA 1.89999998 sec
TD0 1
======== CHANNEL f1 ========
NUC1 13C
P1 27.50 usec
PL1 -1.00 dB
SFO1 100.6298721 MHz
======== CHANNEL f2 ========
CPDPRG2 waltz16
NUC2 1H
PCPD2 100.00 usec
PL2 -3.00 dB
PL12 13.65 dB
PL13 18.00 dB
SFO2 400.1516006 MHz
F2 - Processing parameters
SI 32768
SF 100.6178010 MHz
WDW EM
SSB 0
LB 1.00 Hz
GB 0
PC 1.40
190 180 170 160 150 140 130 120 110 100 90 80 70 60 50 40 30 20 10 0 -10 ppm

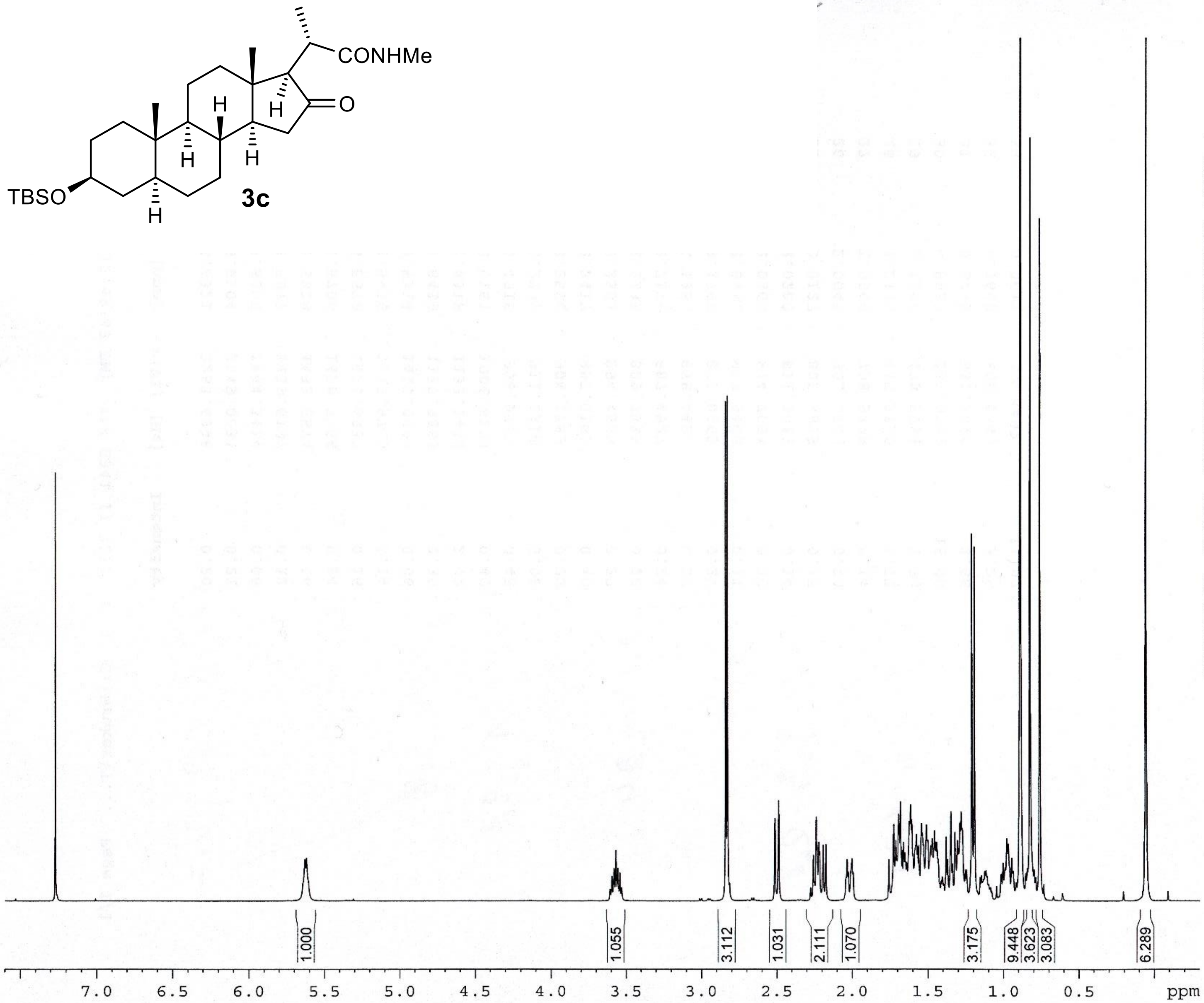

3c


```
Current Data Parameters
NAME        AW G341 II (2)
EXPNO                    1
PROCNO                   1

F2 - Acquisition Parameters
Date_             20200303
Time                 12.52
INSTRUM              spect
PROBHD   5 mm PABBO BB-
PULPROG               zg30
TD                   65536
SOLVENT              CDCl3
NS                     128
DS                       0
SWH              8223.685 Hz
FIDRES           0.125483 Hz
AQ              3.9846387 sec
RG                     322
DW                  60.800 usec
DE                    8.00 usec
TE                   299.2 K
D1              1.00000000 sec
TD0                      1

======== CHANNEL f1 ========
NUC1                    1H
P1                   11.15 usec
PL1                  -3.00 dB
SFO1           400.1524711 MHz

F2 - Processing parameters
SI                   32768
SF             400.1500000 MHz
WDW                     GM
SSB                      0
LB                   -0.20 Hz
GB                     0.2
PC                    1.00
```

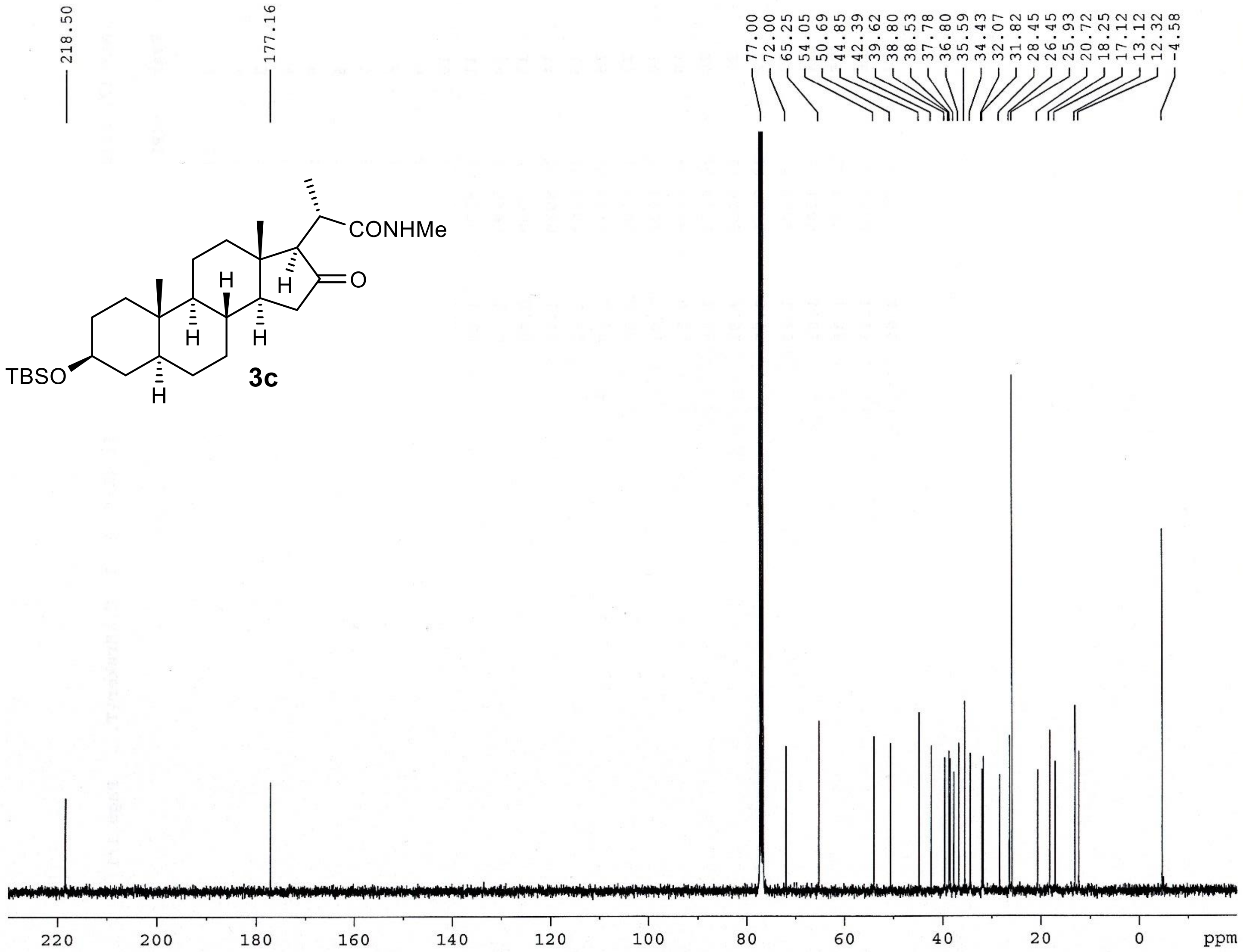


```
Current Data Parameters
NAME      AW G341 II (2)
EXPNO                  2
PROCNO                 1

F2 - Acquisition Parameters
Date_           20200311
Time               17.09
INSTRUM            spect
PROBHD   5 mm PABBO BB-
PULPROG          zgpg30
TD                 65536
SOLVENT            CDCl3
NS                  2816
DS                     4
SWH           32051.281 Hz
FIDRES         0.489064 Hz
AQ            1.0224116 sec
RG                  2050
DW                15.600 usec
DE                  6.00 usec
TE                 296.8 K
D1            2.00000000 sec
d11           0.03000000 sec
DELTA         1.89999998 sec
TD0                    1

======== CHANNEL f1 ========
NUC1                 13C
P1                 27.50 usec
PL1                -1.00 dB
SFO1         100.6298721 MHz

======== CHANNEL f2 ========
CPDPRG2          waltz16
NUC2                  1H
PCPD2             100.00 usec
PL2                -3.00 dB
PL12               13.65 dB
PL13               18.00 dB
SFO2         400.1516006 MHz

F2 - Processing parameters
SI                 32768
SF           100.6177995 MHz
WDW                   EM
SSB                    0
LB                  1.00 Hz
GB                     0
PC                  0.20
```

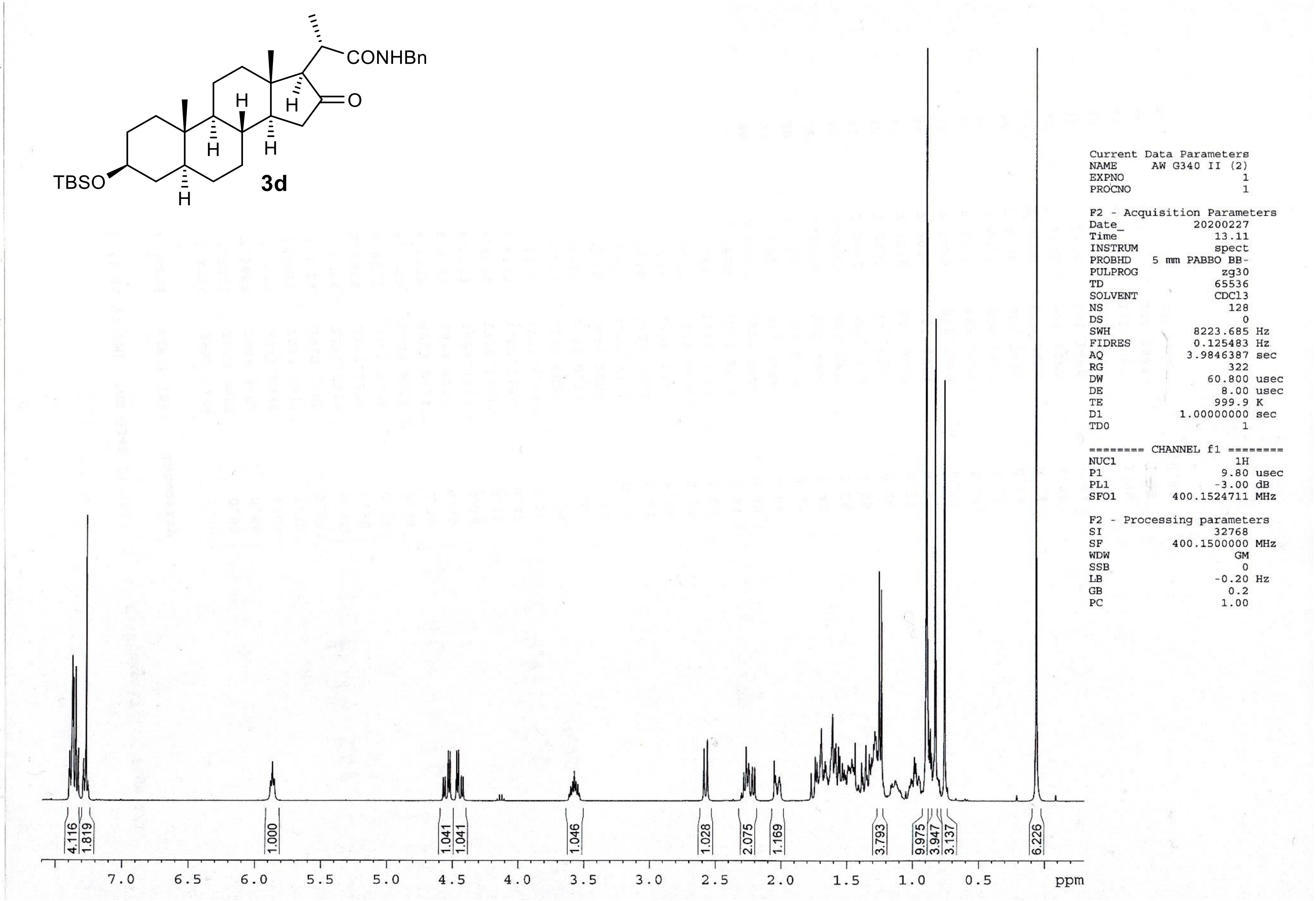

CONHBn
O
H
TBSO
3d
Current Data Parameters
NAME AW G340 II (2)
EXPNO 1
PROCNO 1
F2 - Acquisition Parameters
Date_ 20200227
Time 13.11
INSTRUM spect
PROBHD 5 mm PABBO BB-
PULPROG zg30
TD 65536
SOLVENT CDCl3
NS 128
DS 0
SWH 8223.685 Hz
FIDRES 0.125483 Hz
AQ 3.9846387 sec
RG 322
DW 60.800 usec
DE 8.00 usec
TE 999.9 K
D1 1.00000000 sec
TD0 1
======== CHANNEL f1 ========
NUC1 1H
P1 9.80 usec
PL1 -3.00 dB
SFO1 400.1524711 MHz
F2 - Processing parameters
SI 32768
SF 400.1500000 MHz
WDW GM
SSB 0
LB -0.20 Hz
GB 0.2
PC 1.00
4.116
1.819
1.000
1.041
1.041
1.046
1.028
2.075
1.169
3.793
9.975
3.947
3.137
6.226
7.0 6.5 6.0 5.5 5.0 4.5 4.0 3.5 3.0 2.5 2.0 1.5 1.0 0.5 ppm

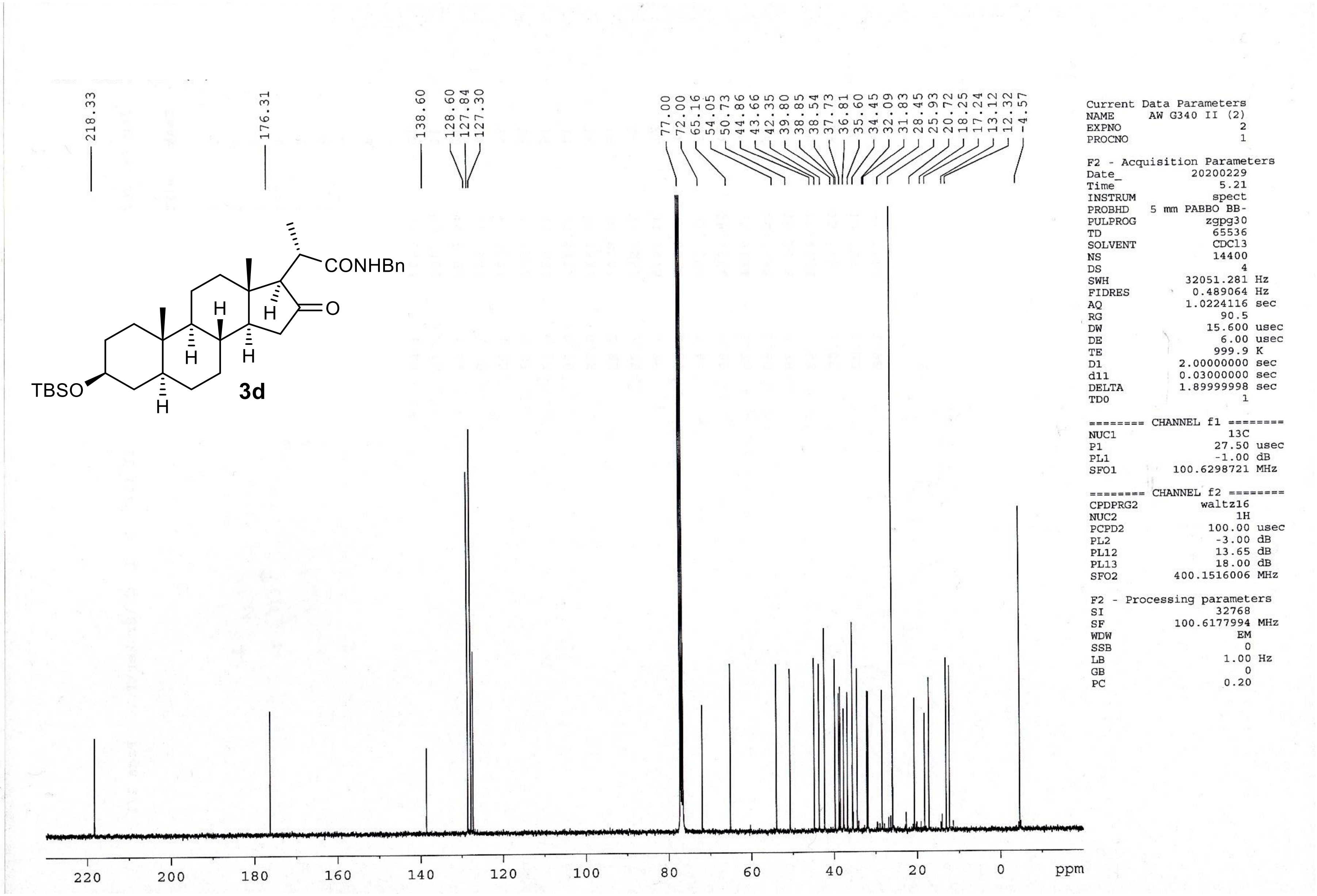
218.33
176.31
138.60
128.60
127.84
127.30
77.00
72.00
65.16
54.05
50.73
44.86
43.66
42.35
39.80
38.85
38.54
37.73
36.81
35.60
34.45
32.09
31.83
28.45
25.93
20.72
18.25
17.24
13.12
12.32
-4.57
CONHBn
O
H
TBSO
3d
Current Data Parameters
NAME AW G340 II (2)
EXPNO 2
PROCNO 1
F2 - Acquisition Parameters
Date_ 20200229
Time 5.21
INSTRUM spect
PROBHD 5 mm PABBO BB-
PULPROG zgpg30
TD 65536
SOLVENT CDCl3
NS 14400
DS 4
SWH 32051.281 Hz
FIDRES 0.489064 Hz
AQ 1.0224116 sec
RG 90.5
DW 15.600 usec
DE 6.00 usec
TE 999.9 K
D1 2.00000000 sec
d11 0.03000000 sec
DELTA 1.89999998 sec
TD0 1
======== CHANNEL f1 ========
NUC1 13C
P1 27.50 usec
PL1 -1.00 dB
SFO1 100.6298721 MHz
======== CHANNEL f2 ========
CPDPRG2 waltz16
NUC2 1H
PCPD2 100.00 usec
PL2 -3.00 dB
PL12 13.65 dB
PL13 18.00 dB
SFO2 400.1516006 MHz
F2 - Processing parameters
SI 32768
SF 100.6177994 MHz
WDW EM
SSB 0
LB 1.00 Hz
GB 0
PC 0.20
220 200 180 160 140 120 100 80 60 40 20 0 ppm

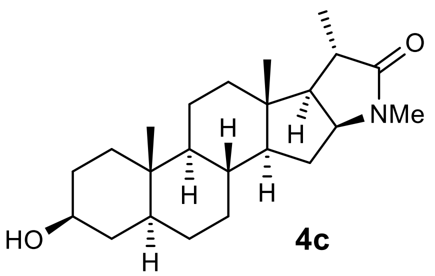

HO
H
NMe
O

4c

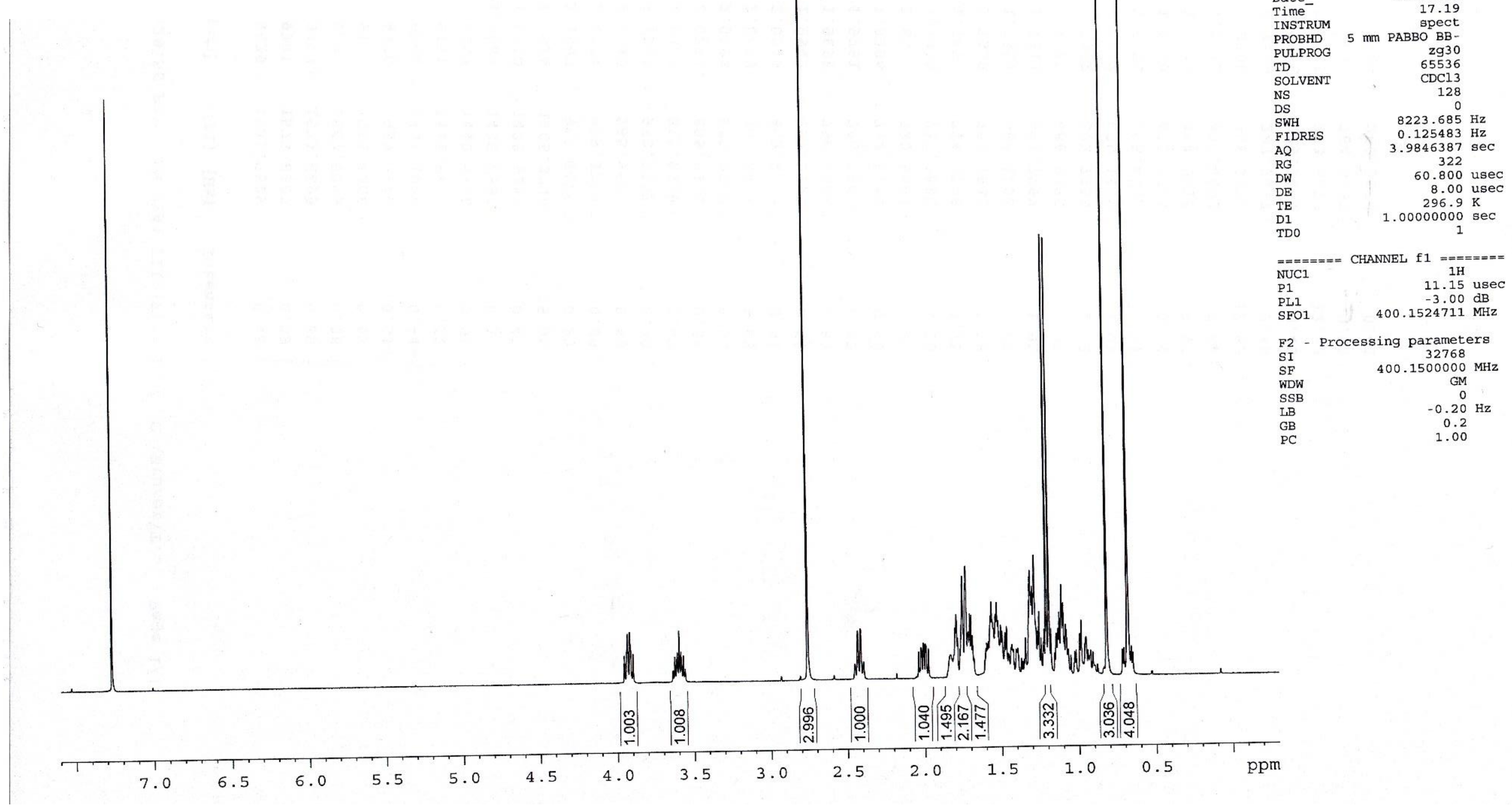

Current Data Parameters
NAME AW G341 III (2)
EXPNO 1
PROCNO 1
F2 - Acquisition Parameters
Date_ 20200304
Time 17.19
INSTRUM spect
PROBHD 5 mm PABBO BB-
PULPROG zg30
TD 65536
SOLVENT CDCl3
NS 128
DS 0
SWH 8223.685 Hz
FIDRES 0.125483 Hz
AQ 3.9846387 sec
RG 322
DW 60.800 usec
DE 8.00 usec
TE 296.9 K
D1 1.00000000 sec
TD0 1
======== CHANNEL f1 ========
NUC1 1H
P1 11.15 usec
PL1 -3.00 dB
SFO1 400.1524711 MHz
F2 - Processing parameters
SI 32768
SF 400.1500000 MHz
WDW GM
SSB 0
LB -0.20 Hz
GB 0.2
PC 1.00
1.003
1.008
2.996
1.000
1.040
1.495
2.167
1.477
3.332
3.036
4.048
7.0 6.5 6.0 5.5 5.0 4.5 4.0 3.5 3.0 2.5 2.0 1.5 1.0 0.5 ppm

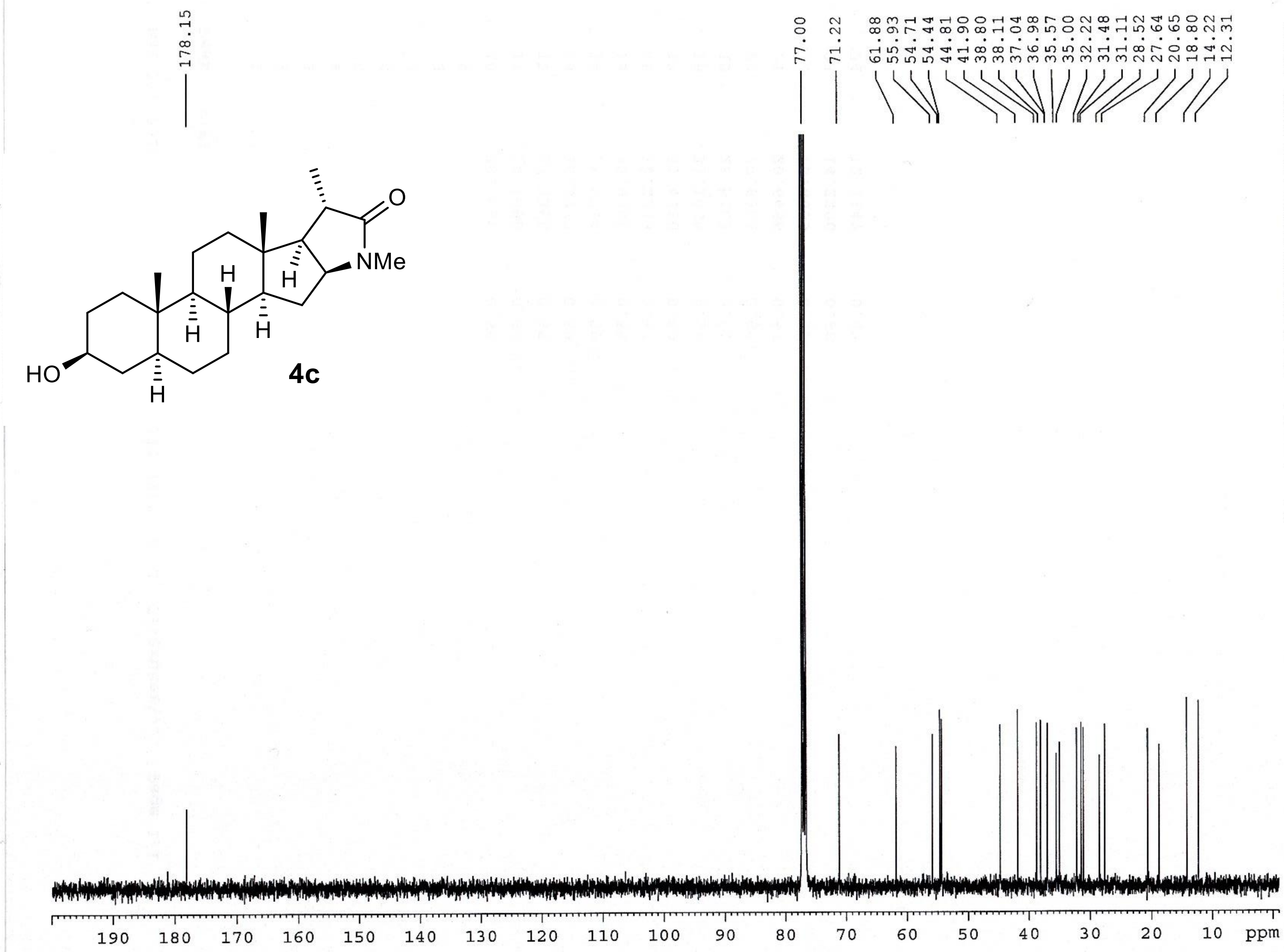

Current Data Parameters	
NAME	AW G341 III (2)
EXPNO	2
PROCNO	1

F2 - Acquisition Parameters	
Date_	20200310
Time	14.58
INSTRUM	spect
PROBHD	5 mm PABBO BB-
PULPROG	zgpg30
TD	65536
SOLVENT	CDCl3
NS	1920
DS	4
SWH	32051.281 Hz
FIDRES	0.489064 Hz
AQ	1.0224116 sec
RG	2050
DW	15.600 usec
DE	6.00 usec
TE	297.0 K
D1	2.00000000 sec
d11	0.03000000 sec
DELTA	1.89999998 sec
TD0	1

======== CHANNEL f1 ========	
NUC1	13C
P1	27.50 usec
PL1	-1.00 dB
SFO1	100.6298721 MHz

======== CHANNEL f2 ========	
CPDPRG2	waltz16
NUC2	1H
PCPD2	100.00 usec
PL2	-3.00 dB
PL12	13.65 dB
PL13	18.00 dB
SFO2	400.1516006 MHz

F2 - Processing parameters	
SI	32768
SF	100.6177993 MHz
WDW	EM
SSB	0
LB	1.00 Hz
GB	0
PC	0.20

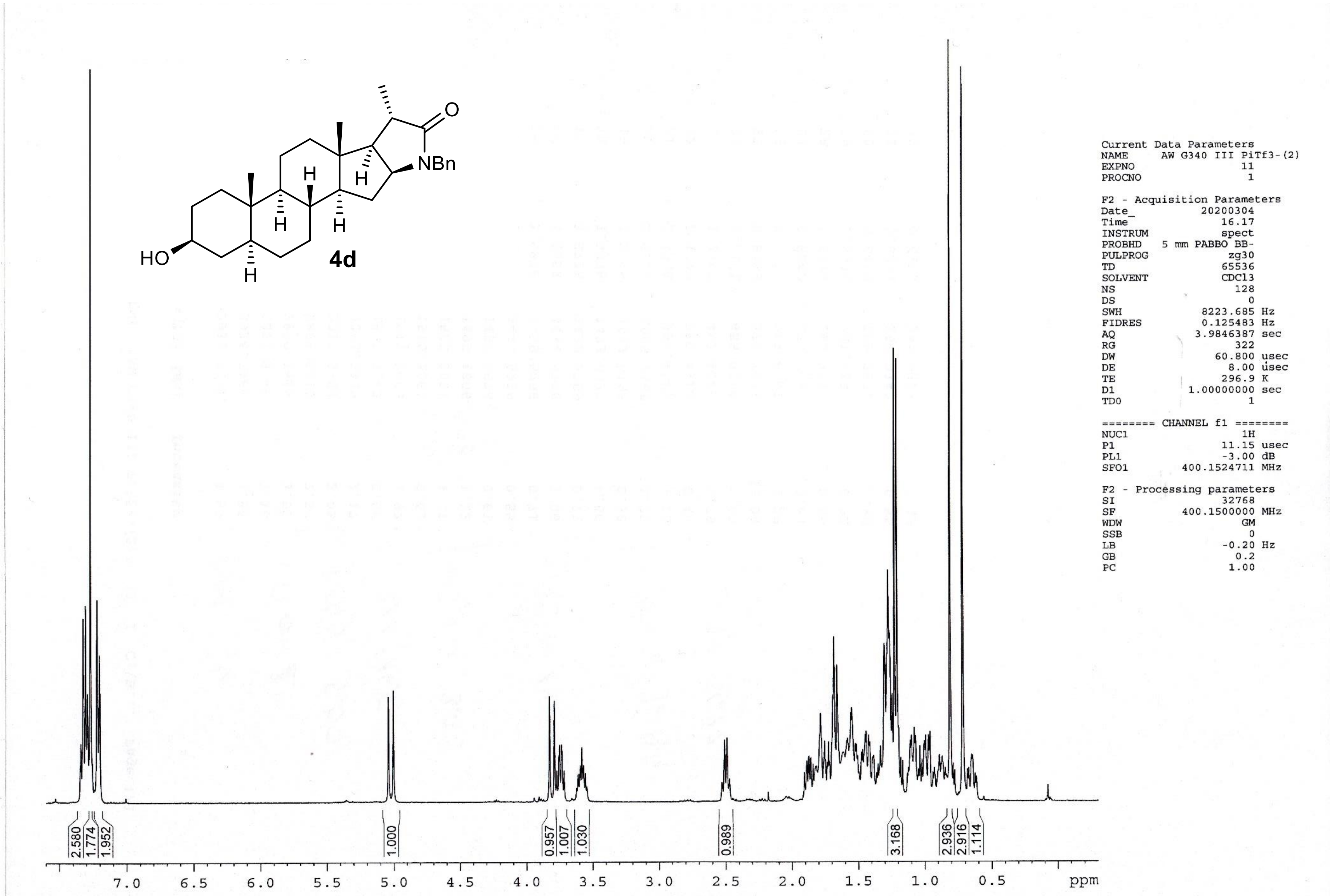
O
NBn
H
H
H
H
HO
H
4d
Current Data Parameters
NAME AW G340 III PiTf3-(2)
EXPNO 11
PROCNO 1
F2 - Acquisition Parameters
Date_ 20200304
Time_ 16.17
INSTRUM spect
PROBHD 5 mm PABBO BB-
PULPROG zg30
TD 65536
SOLVENT CDCl3
NS 128
DS 0
SWH 8223.685 Hz
FIDRES 0.125483 Hz
AQ 3.9846387 sec
RG 322
DW 60.800 usec
DE 8.00 usec
TE 296.9 K
D1 1.00000000 sec
TD0 1
======== CHANNEL f1 ========
NUC1 1H
P1 11.15 usec
PL1 -3.00 dB
SFO1 400.1524711 MHz
F2 - Processing parameters
SI 32768
SF 400.1500000 MHz
WDW GM
SSB 0
LB -0.20 Hz
GB 0.2
PC 1.00
2.580
1.774
1.952
1.000
0.957
1.007
1.030
0.989
3.168
2.936
2.916
1.114
7.0
6.5
6.0
5.5
5.0
4.5
4.0
3.5
3.0
2.5
2.0
1.5
1.0
0.5
ppm

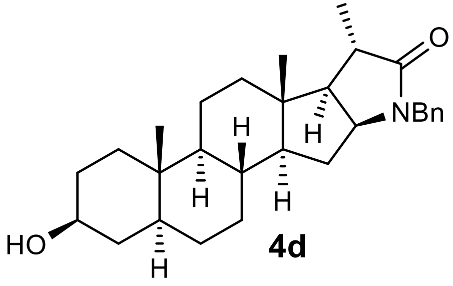

4d

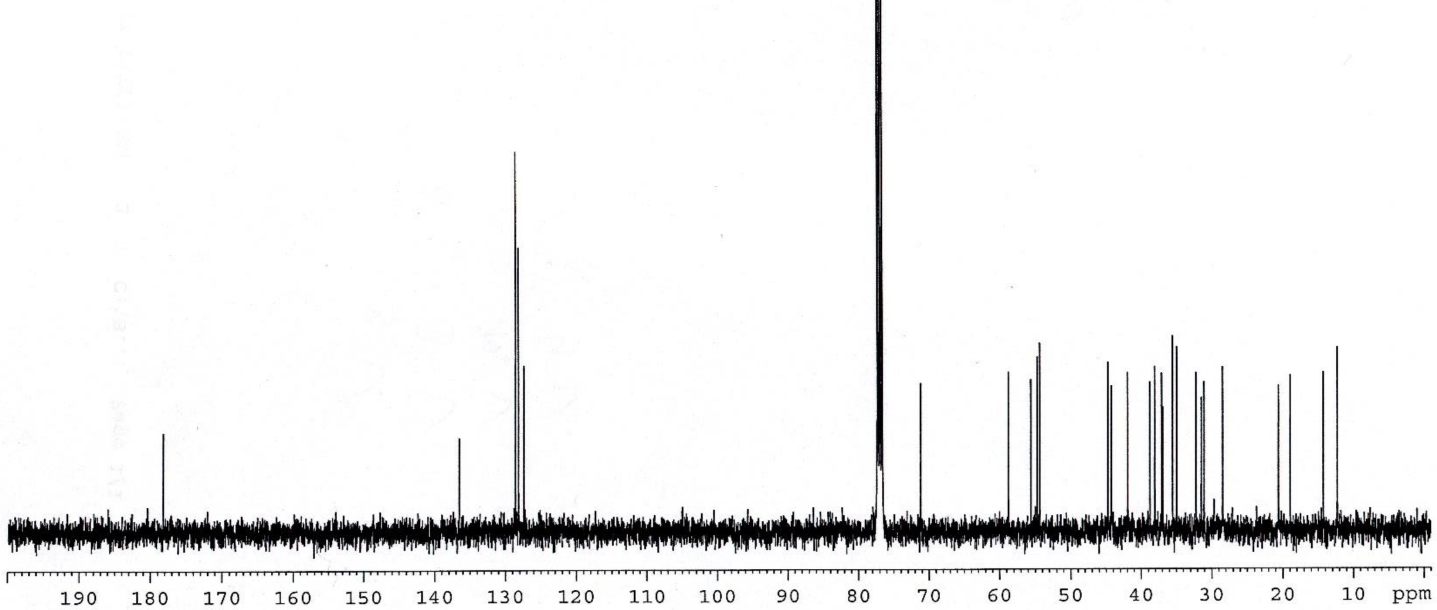


```
Current Data Parameters
NAME     AW G340 III PiTf3-(2)
EXPNO                        2
PROCNO                       1

F2 - Acquisition Parameters
Date_               20200305
Time                   10.35
INSTRUM                spect
PROBHD   5 mm PABBO BB-
PULPROG               zgpg30
TD                     65536
SOLVENT                CDCl3
NS                      1664
DS                         4
SWH             32051.281 Hz
FIDRES           0.489064 Hz
AQ              1.0224116 sec
RG                      2050
DW                15.600 usec
DE                  6.00 usec
TE                  296.9 K
D1            2.00000000 sec
d11           0.03000000 sec
DELTA         1.89999998 sec
TD0                        1

======== CHANNEL f1 ========
NUC1                     13C
P1                 27.50 usec
PL1                 -1.00 dB
SFO1         100.6298721 MHz

======== CHANNEL f2 ========
CPDPRG2              waltz16
NUC2                      1H
PCPD2             100.00 usec
PL2                 -3.00 dB
PL12                13.65 dB
PL13                18.00 dB
SFO2         400.1516006 MHz

F2 - Processing parameters
SI                     32768
SF           100.6177994 MHz
WDW                       EM
SSB                        0
LB                   1.00 Hz
GB                         0
PC                      0.20
```

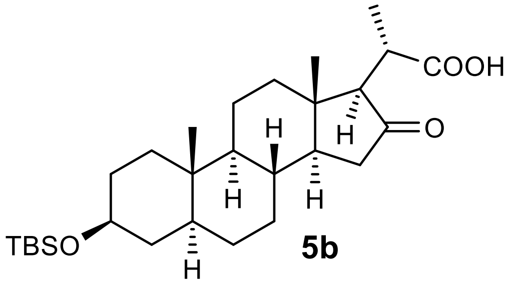

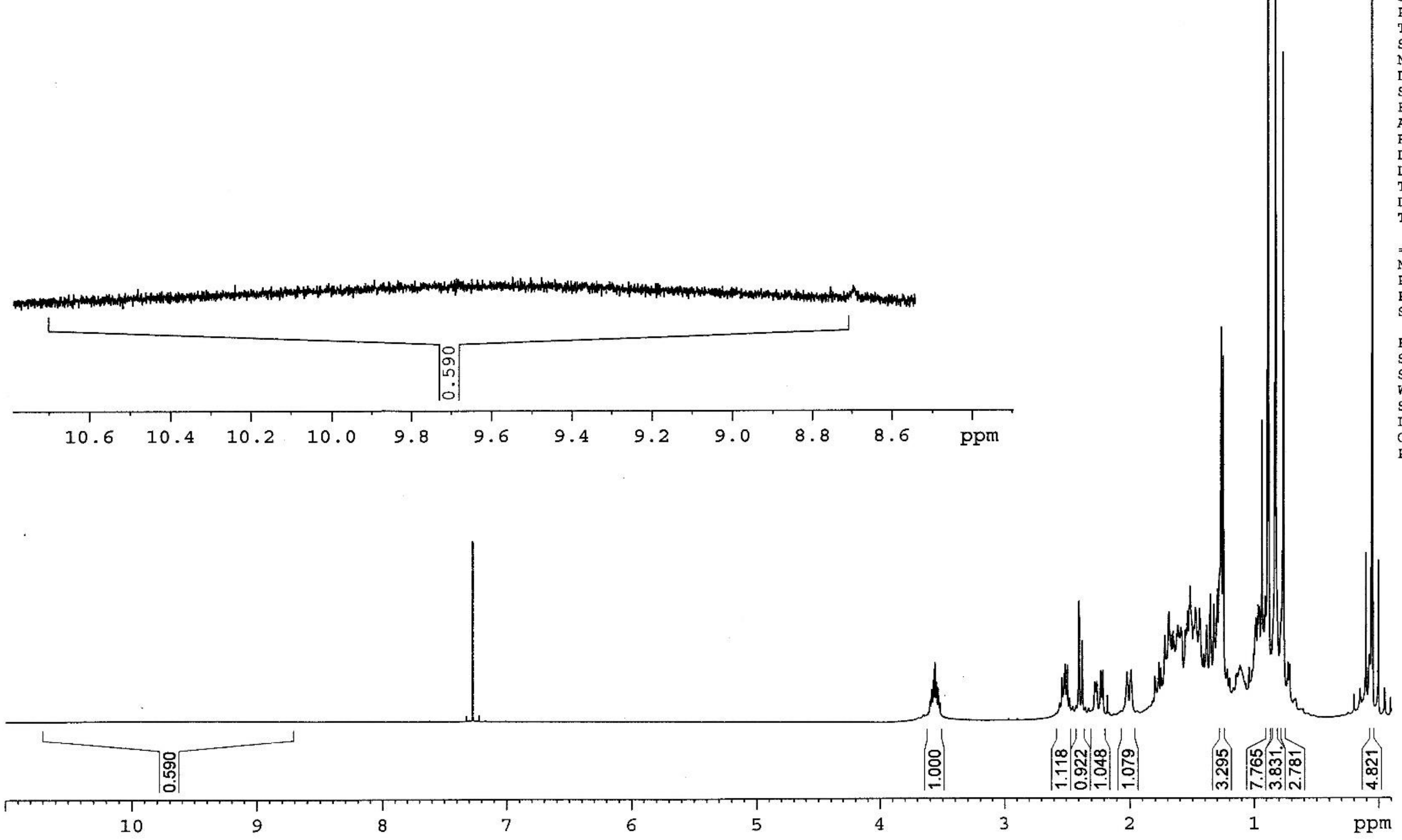


```
Current Data Parameters
NAME      DP 30TBS-oksokwas
EXPNO                     1
PROCNO                    1

F2 - Acquisition Parameters
Date_              20200111
Time                  16.36
INSTRUM               spect
PROBHD     5 mm PABBO BB-
PULPROG                zg30
TD                    65536
SOLVENT               CDCl3
NS                      128
DS                        0
SWH                8223.685 Hz
FIDRES             0.125483 Hz
AQ                3.9846387 sec
RG                      228
DW                   60.800 usec
DE                     8.00 usec
TE                    999.9 K
D1               1.00000000 sec
TD0                       1

======== CHANNEL f1 ========
NUC1                     1H
P1                    11.15 usec
PL1                   -3.00 dB
SFO1            400.1524711 MHz

F2 - Processing parameters
SI                    32768
SF              400.1500000 MHz
WDW                      GM
SSB                       0
LB                    -0.20 Hz
GB                      0.2
PC                     1.00
```

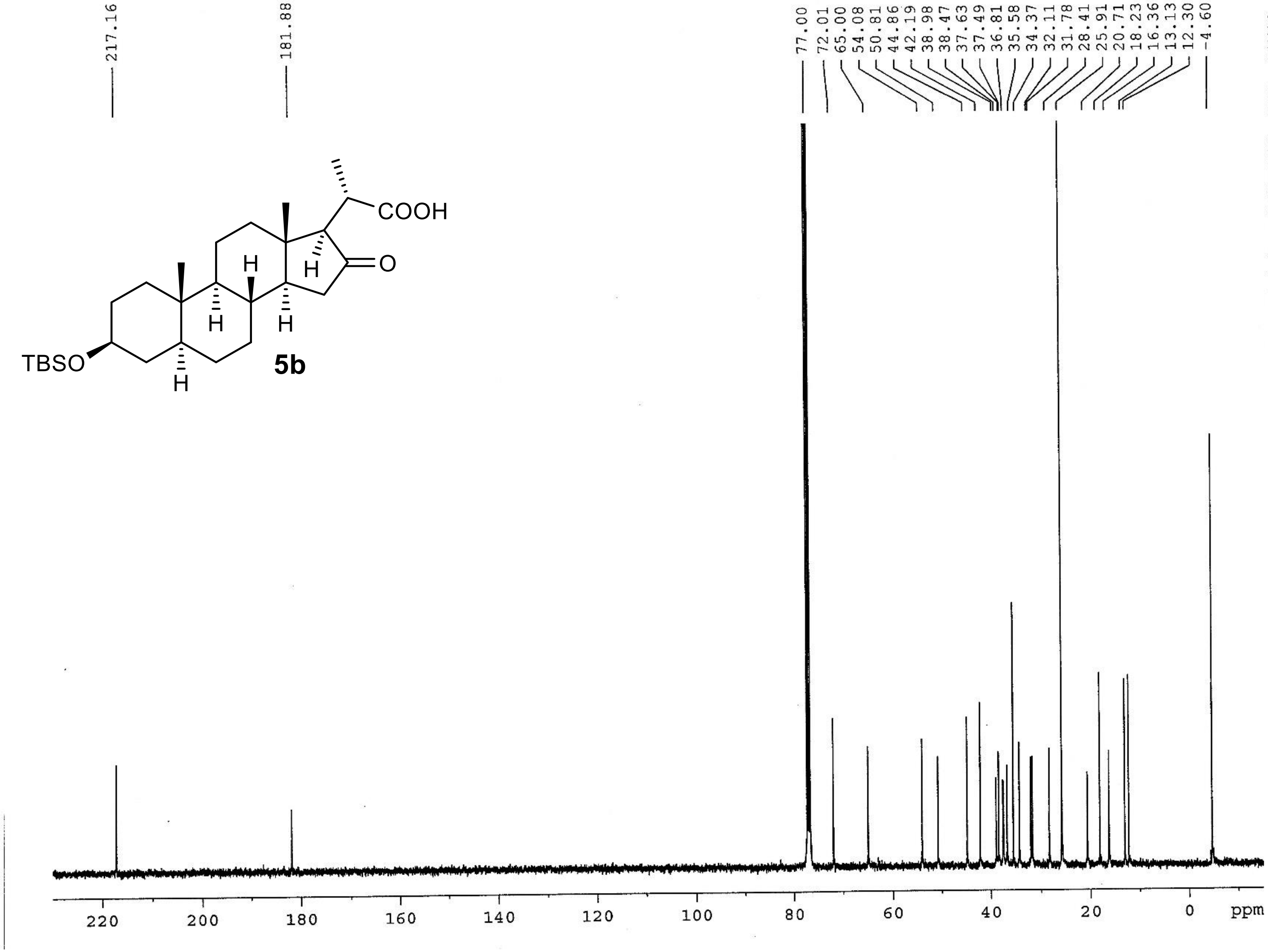

5b

```
Current Data Parameters
NAME        DP 30TBS-oksokwas
EXPNO                       2
PROCNO                      1

F2 - Acquisition Parameters
Date_                20200111
Time                    16.44
INSTRUM                 spect
PROBHD     5 mm PABBO BB-
PULPROG               zgpg30
TD                      65536
SOLVENT                 CDCl3
NS                       1920
DS                          4
SWH             32051.281 Hz
FIDRES           0.489064 Hz
AQ              1.0224116 sec
RG                       2050
DW                 15.600 usec
DE                   6.00 usec
TE                    999.9 K
D1             2.00000000 sec
d11            0.03000000 sec
DELTA          1.89999998 sec
TD0                         1

======== CHANNEL f1 ========
NUC1                      13C
P1                  27.50 usec
PL1                  -1.00 dB
SFO1          100.6298721 MHz

======== CHANNEL f2 ========
CPDPRG2               waltz16
NUC2                       1H
PCPD2              100.00 usec
PL2                  -3.00 dB
PL12                 13.65 dB
PL13                 18.00 dB
SFO2          400.1516006 MHz

F2 - Processing parameters
SI                      32768
SF            100.6178015 MHz
WDW                        EM
SSB                         0
LB                    1.00 Hz
GB                          0
PC                       0.20
```

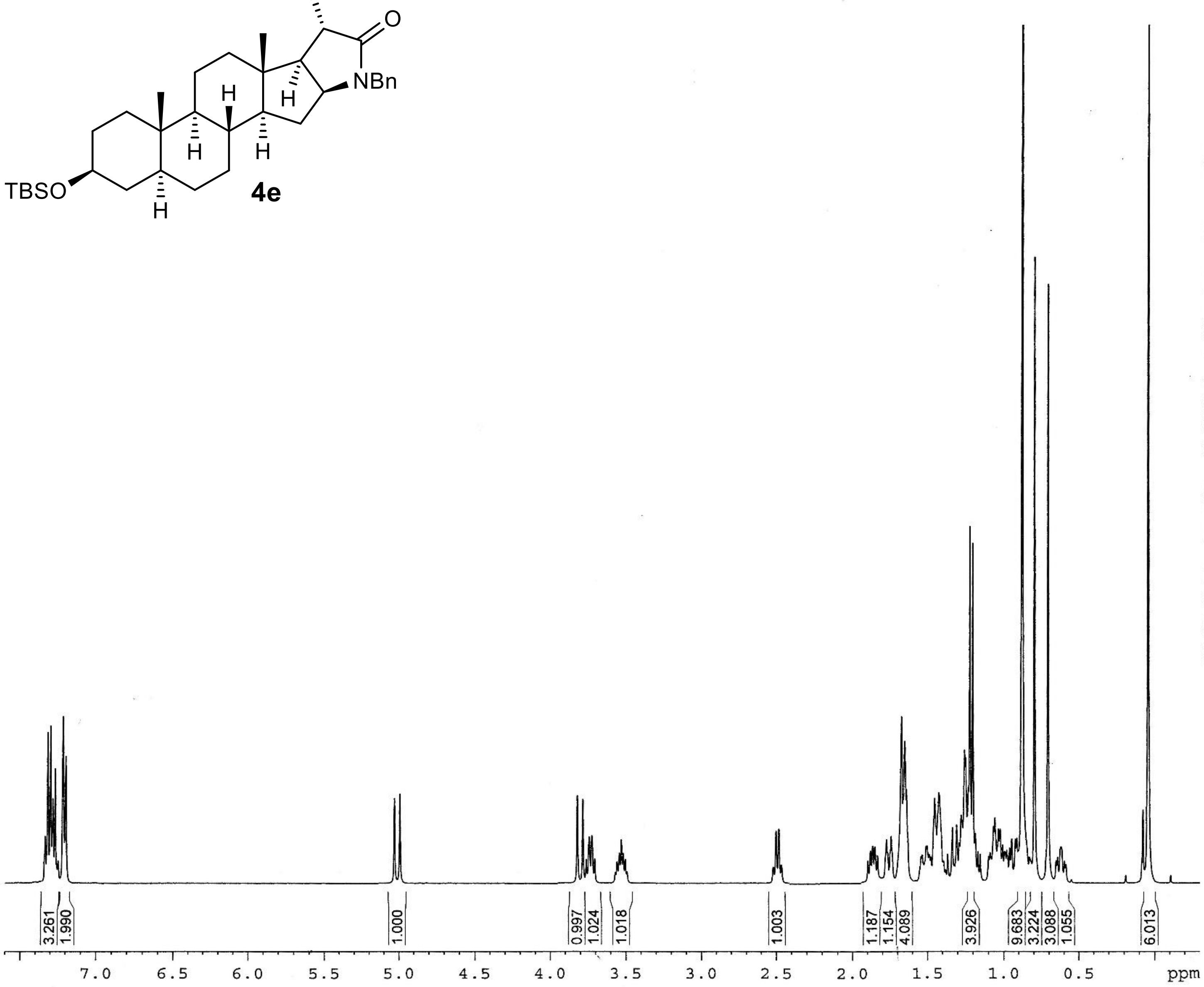


```
Current Data Parameters
NAME             AB 601D-z3
EXPNO                     1
PROCNO                    1

F2 - Acquisition Parameters
Date_              20200304
Time                  13.46
INSTRUM               spect
PROBHD   5 mm PABBO BB-
PULPROG                zg30
TD                    65536
SOLVENT               CDCl3
NS                       64
DS                        0
SWH             8223.685 Hz
FIDRES          0.125483 Hz
AQ             3.9846387 sec
RG                      256
DW               60.800 usec
DE                 8.00 usec
TE                  296.9 K
D1           1.00000000 sec
TD0                       1

======== CHANNEL f1 ========
NUC1                     1H
P1                11.15 usec
PL1                -3.00 dB
SFO1         400.1524711 MHz

F2 - Processing parameters
SI                    32768
SF           400.1500008 MHz
WDW                      GM
SSB                       0
LB                 -0.20 Hz
GB                      0.2
PC                     1.00
```

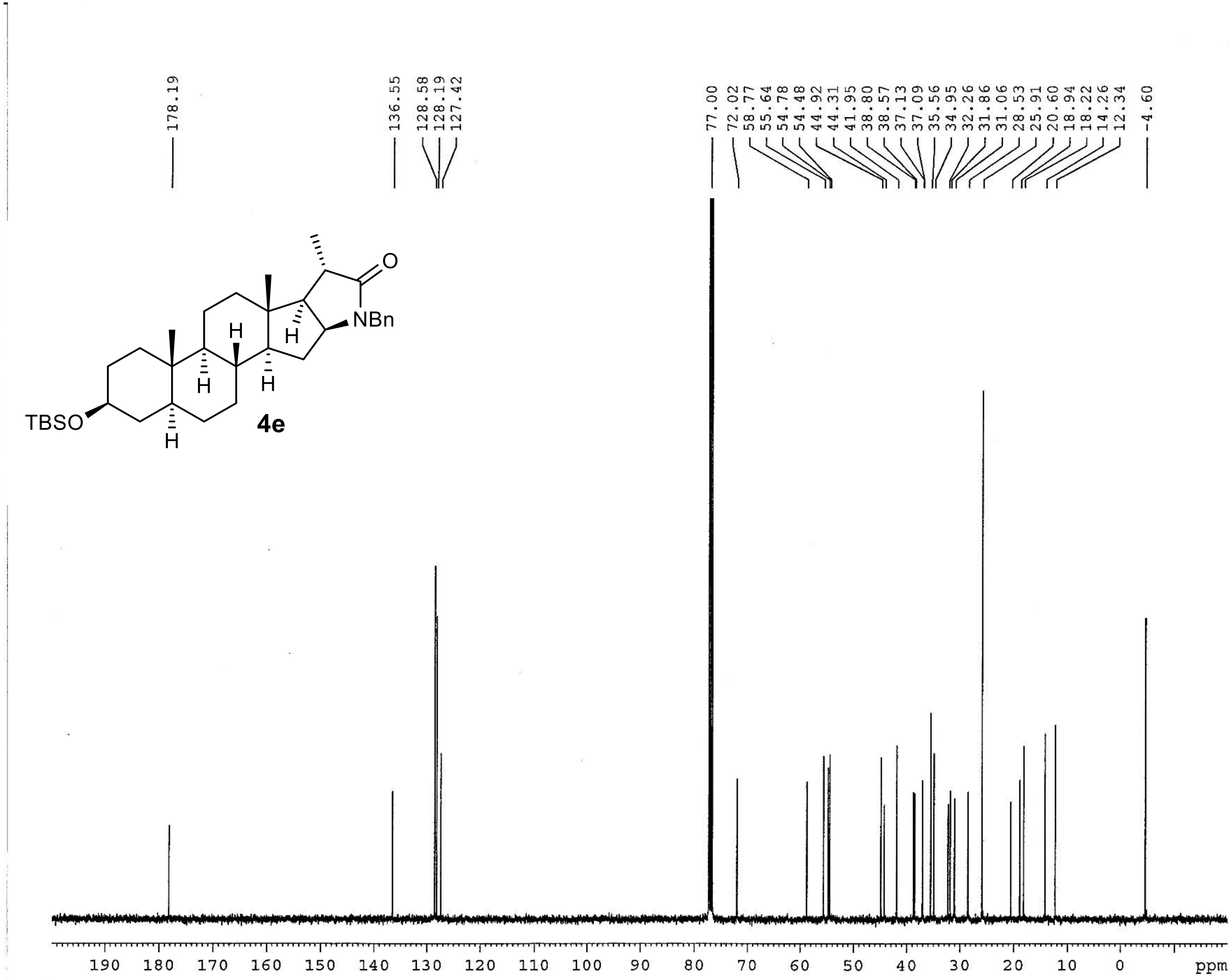


```
Current Data Parameters
NAME          AB 601D-z3
EXPNO                  2
PROCNO                 1

F2 - Acquisition Parameters
Date_           20200306
Time               18.46
INSTRUM            spect
PROBHD   5 mm PABBO BB-
PULPROG           zgpg30
TD                 65536
SOLVENT            CDCl3
NS                   768
DS                     4
SWH            32051.281 Hz
FIDRES          0.489064 Hz
AQ             1.0224116 sec
RG                  2050
DW                15.600 usec
DE                  6.00 usec
TE                 297.0 K
D1            2.00000000 sec
d11           0.03000000 sec
DELTA         1.89999998 sec
TD0                    1

======== CHANNEL f1 ========
NUC1                 13C
P1                 27.50 usec
PL1                -1.00 dB
SFO1         100.6298721 MHz

======== CHANNEL f2 ========
CPDPRG2          waltz16
NUC2                  1H
PCPD2             100.00 usec
PL2                -3.00 dB
PL12               13.65 dB
PL13               18.00 dB
SFO2         400.1516006 MHz

F2 - Processing parameters
SI                 32768
SF           100.6178015 MHz
WDW                   EM
SSB                    0
LB                  1.00 Hz
GB                     0
PC                  1.40
```

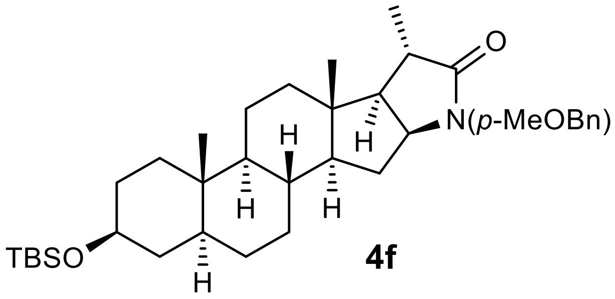

4f

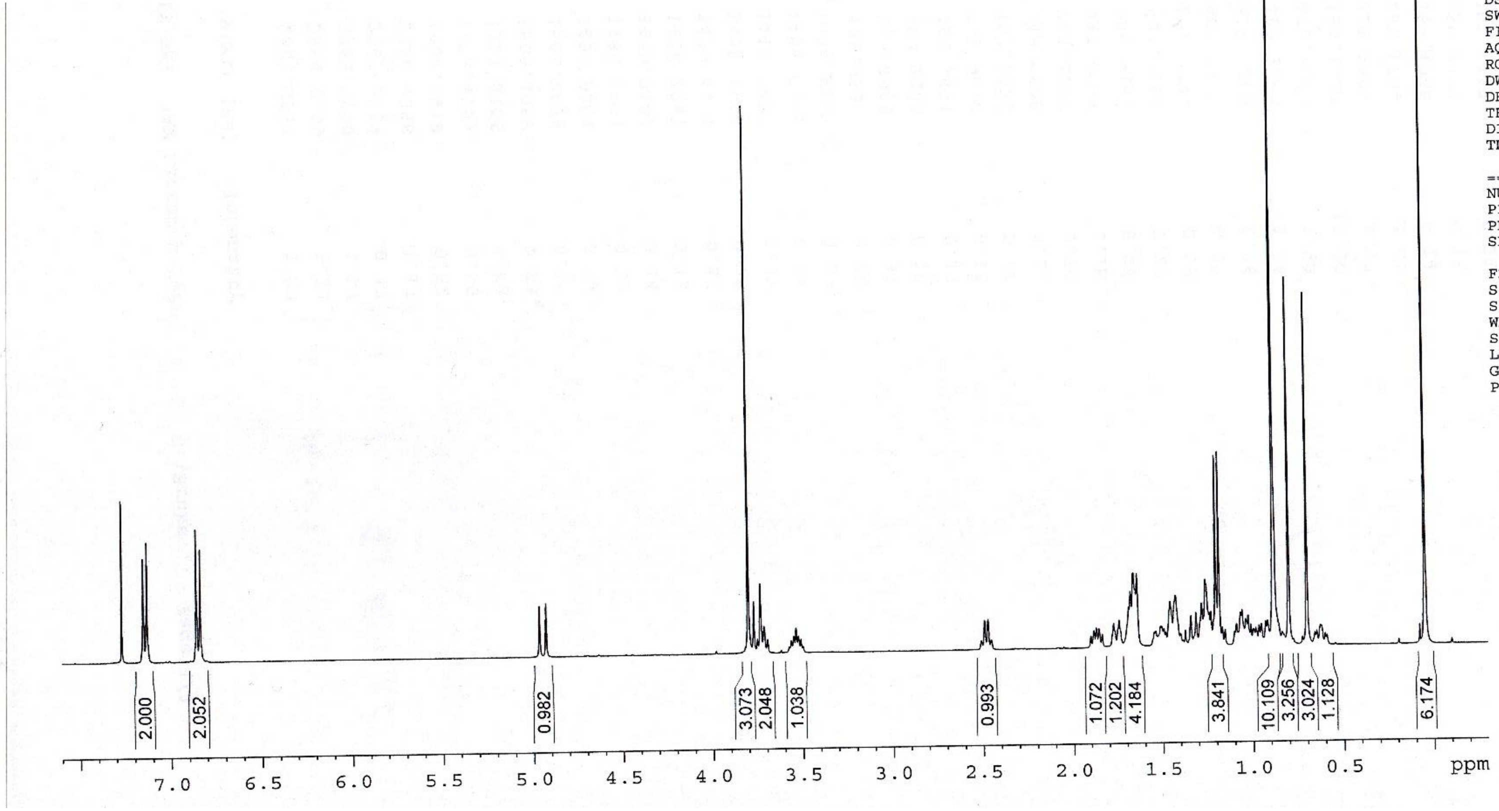


```
Current Data Parameters
NAME      AW Lactam p-MeBn
EXPNO                    1
PROCNO                   1

F2 - Acquisition Parameters
Date_             20200327
Time                 11.54
INSTRUM              spect
PROBHD   5 mm PABBO BB-
PULPROG               zg30
TD                   65536
SOLVENT              CDC13
NS                     128
DS                       0
SWH               8223.685 Hz
FIDRES            0.125483 Hz
AQ               3.9846387 sec
RG                     161
DW                  60.800 usec
DE                    8.00 usec
TE                   999.9 K
D1              1.00000000 sec
TD0                      1

======== CHANNEL f1 ========
NUC1                    1H
P1                   11.15 usec
PL1                  -3.00 dB
SFO1           400.1524711 MHz

F2 - Processing parameters
SI                   32768
SF             400.1500000 MHz
WDW                     GM
SSB                      0
LB                   -0.20 Hz
GB                     0.2
PC                    1.00
```

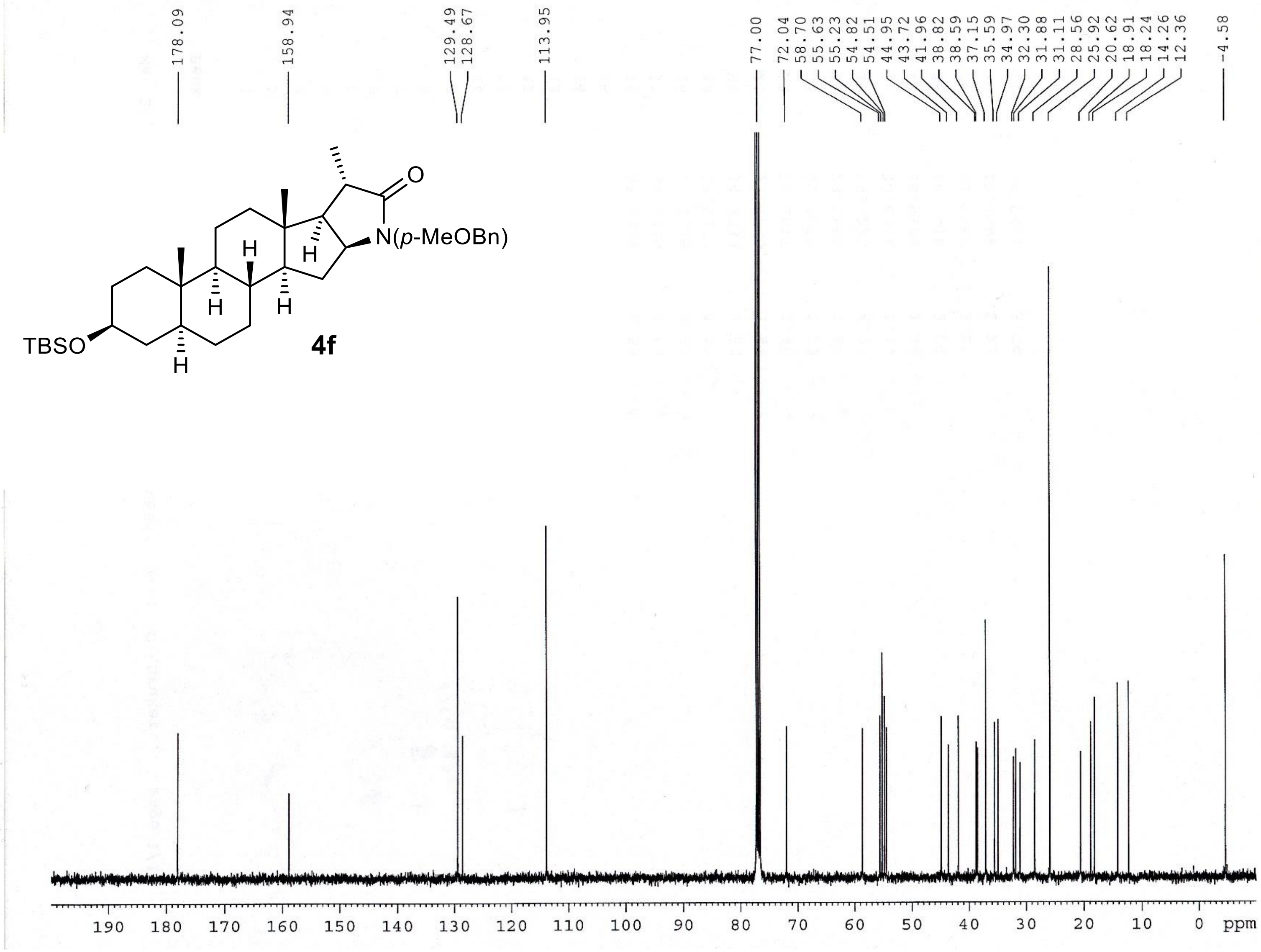


```
Current Data Parameters
NAME       AW Lactam p-MeBn
EXPNO                     2
PROCNO                    1

F2 - Acquisition Parameters
Date_              20200327
Time                  12.30
INSTRUM               spect
PROBHD     5 mm PABBO BB-
PULPROG              zgpg30
TD                    65536
SOLVENT               CDCl3
NS                     2240
DS                        4
SWH           32051.281 Hz
FIDRES         0.489064 Hz
AQ            1.0224116 sec
RG                     2050
DW               15.600 usec
DE                 6.00 usec
TE                  999.9 K
D1           2.00000000 sec
d11          0.03000000 sec
DELTA        1.89999998 sec
TD0                       1

======== CHANNEL f1 ========
NUC1                    13C
P1                27.50 usec
PL1               -1.00 dB
SFO1        100.6298721 MHz

======== CHANNEL f2 ========
CPDPRG2             waltz16
NUC2                     1H
PCPD2            100.00 usec
PL2               -3.00 dB
PL12              13.65 dB
PL13              18.00 dB
SFO2        400.1516006 MHz

F2 - Processing parameters
SI                    32768
SF          100.6177997 MHz
WDW                      EM
SSB                       0
LB                 1.00 Hz
GB                        0
PC                     0.20
```
